# Predictive Coding of Dynamical Variables in Balanced Spiking Networks

**DOI:** 10.1371/journal.pcbi.1003258

**Published:** 2013-11-14

**Authors:** Martin Boerlin, Christian K. Machens, Sophie Denève

**Affiliations:** 1 Group for Neural Theory, Département d'Études Cognitives, École Normale Supérieure, Paris, France; 2 Champalimaud Neuroscience Programme, Champalimaud Centre for the Unknown, Lisbon, Portugal; Indiana University, United States of America

## Abstract

Two observations about the cortex have puzzled neuroscientists for a long time. First, neural responses are highly variable. Second, the level of excitation and inhibition received by each neuron is tightly balanced at all times. Here, we demonstrate that both properties are necessary consequences of neural networks that represent information efficiently in their spikes. We illustrate this insight with spiking networks that represent dynamical variables. Our approach is based on two assumptions: We assume that information about dynamical variables can be read out linearly from neural spike trains, and we assume that neurons only fire a spike if that improves the representation of the dynamical variables. Based on these assumptions, we derive a network of leaky integrate-and-fire neurons that is able to implement arbitrary linear dynamical systems. We show that the membrane voltage of the neurons is equivalent to a prediction error about a common population-level signal. Among other things, our approach allows us to construct an integrator network of spiking neurons that is robust against many perturbations. Most importantly, neural variability in our networks cannot be equated to noise. Despite exhibiting the same single unit properties as widely used population code models (e.g. tuning curves, Poisson distributed spike trains), balanced networks are orders of magnitudes more reliable. Our approach suggests that spikes do matter when considering how the brain computes, and that the reliability of cortical representations could have been strongly underestimated.

## Author Summary

Two observations about the cortex have puzzled and fascinated neuroscientists for a long time. First, neural responses are highly variable. Second, the level of excitation and inhibition received by each neuron is tightly balanced at all times. Here, we demonstrate that both properties are necessary consequences of neural networks representing information reliably and with a small number of spikes. To achieve such efficiency, spikes of individual neurons must communicate prediction errors about a common population-level signal, automatically resulting in balanced excitation and inhibition and highly variable neural responses. We illustrate our approach by focusing on the implementation of linear dynamical systems. Among other things, this allows us to construct a network of spiking neurons that can integrate input signals, yet is robust against many perturbations. Most importantly, our approach shows that neural variability cannot be equated to noise. Despite exhibiting the same single unit properties as other widely used network models, our balanced networks are orders of magnitudes more reliable. Our results suggest that the precision of cortical representations has been strongly underestimated.

## Introduction

Neural systems need to integrate, store, and manipulate sensory information before acting upon it. Various neurophysiological and psychophysical experiments have provided examples of how these feats are accomplished in the brain, from the integration of sensory stimuli to decision-making [1], from the short-term storage of information [2] to the generation of movement sequences [3]. At the same time, it has been far more difficult to pin down the precise mechanisms underlying these functions.

A lot of research on neural mechanisms has focused on studying neural networks in the framework of attractor dynamics [4]–[6]. These models generally assume that the system's state variables are represented by the instantaneous firing rates of neurons. While quite successful in reproducing some features of electrophysiological data, these models have had a hard time reproducing the irregular, Poisson-like statistics of cortical spike trains. A common assumption is that the random nature of spike times is averaged out over larger populations of neurons or longer periods of time [7]–[10]. However, the biophysical sources of noise in individual neurons are insufficient to explain such variability [11]–[13].

Several researchers have therefore suggested that irregular spike timing arises as a consequence of network dynamics [8], [14]. Indeed, large networks of leaky integrate-and-fire (LIF) neurons with balanced excitation and inhibition can be “chaotic” and generate asynchronous and Poisson-like firing statistics [15]–[18]. While these studies explain how relatively deterministic single units can generate similar statistical properties as random spike generators in rate models, they generally do not clarify how particular computations can be carried out, nor do they fundamentally answer why the brain would be operating in such a regime.

Here we show that the properties of balanced networks can be derived from a single efficiency principle, which in turn allows us to design balanced networks that perform a wide variety of computations. We start from the assumption that dynamical variables are encoded such that they can be extracted from output spike trains by simple synaptic integration. We then specify a loss function that measures the system's performance with respect to an idealized dynamical system. We prescribe that neurons should only fire a spike if that decreases the loss function. From these assumptions, we derive a recurrent network of LIF neurons that is able to implement any linear dynamical system. We show that neurons in our network track a prediction error in their membrane potential and only fire a spike if that prediction error exceeds a certain value, a form of predictive coding.

Our work shows how the ideas of predictive coding with spikes, first laid out within a Bayesian framework [19], 20, can be generalized to design spiking neural networks that implement arbitrary linear dynamical systems. Such multivariate dynamical systems are quite powerful and have remained a mainstay of control-engineering for real-world systems [21]. Importantly, the networks maintain a tight balance between the excitatory and inhibitory currents received by each unit, as has been reported at several levels of cortical processing [22]–[26]. The spike trains are asynchronous and irregular. However, this variability is not noise: The neural population essentially acts as a deterministic “super-unit”, tracking the variable with quasi-perfect accuracy while each individual neuron appears to behave stochastically. We illustrate our approach and its usefulness with several biologically relevant examples.

## Results

### Assumptions

**Figure 1:**
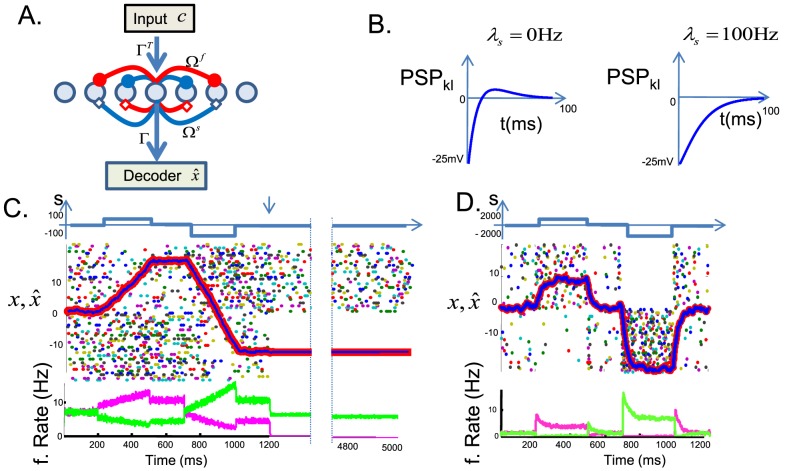
Spike-based implementation of linear dynamical systems. (A) Structure of the network: the neurons receive an input 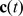, scaled by feedforward weights 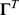, which is internally processed through fast and slow recurrent connections, 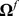 and 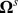, to yield firing rates that can be read out by a linear decoder with weights 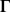 to yield estimates of the dynamical variables, 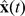. Connections: red, excitatory; blue, inhibitory; filled circle endpoints, fast; empty diamond endpoints, slow. (B) Exemplary, effective postsynaptic potentials between neurons from two different networks. (C) Sensory integrator network for 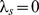 (perfect integrator). Top panel: Sensory stimulus 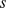 (blue line). Before 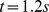, the neurons integrate a slightly noisy version of the stimulus, 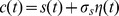, where 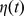 is unit-variance Gaussian noise. At 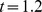
s (downward pointing arrow) all inputs to the network cease (i.e. 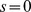, 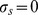). Middle panel: Raster plot of 140 model units for a given trial. Top 70 neurons have negative kernels (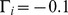), and bottom 70 neurons have positive kernels (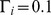). Each dot represents a spike. Thin blue line: state 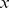. Thick red line: Network estimate 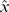. Bottom panel: Mean firing rate (over 500 presentations of identical stimuli 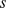, but with different instantiations of the sensory noise 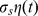) for the population of neurons with positive kernels (magenta) or negative kernels (green). (D) Same as C but for 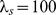
Hz. Parameters in A–D: 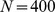, 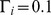 for 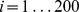, 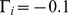 for 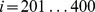, 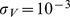, 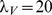
Hz, 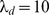
Hz, 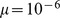, 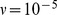, 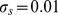 (in C) and 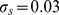 (in D). Simulation time step (Euler method) 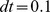
msec. The noise parameters, 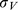 and 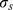, represent the standard deviation of the noise injected in each 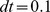
ms time step.

Our basic model strategy is represented in Fig. 1 A. Let us consider a linear dynamical system describing the temporal evolution of a vector of 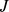 dynamical variables, 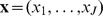:
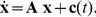
(1)Here 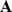 is the state transition matrix, and 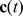 are time-varying, external inputs or command variables. We want to build a neural network composed of 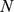 neurons, taking initial state 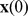 and commands 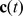 as inputs, and reproducing the temporal trajectory of 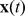. Specifically, we want to be able to read an estimate 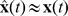 of the dynamical variable from the network's spike trains 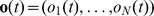. These output spike trains are given by 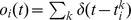, where 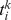 is the time of the 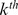 spike in neuron 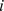.

Our first assumption is that the estimate 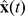 is obtained by a weighted, leaky integration of the spike trains,
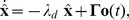
(2)where the 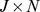 matrix 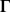 contains the decoding or output weights of all neurons, and 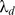 is the read-out's decay rate. Whenever neuron 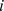 fires, a 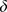-function is added to its spike train, 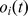. The integration of the respective delta-function contributes a decaying exponential kernel, 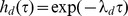, weighted by 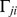, to each dynamical variable, 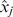. This contribution can be interpreted as a simplified postsynaptical potential (PSP). The effect of a neuron's spike can be summarized by its weights, 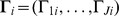, which we call the output kernel of neuron 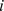. Note that these weights correspond to the columns of the matrix 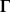. The estimate 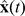 can also be written as a weighted linear summation of the neuron's firing rates, 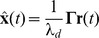, if we define the time-varying firing rates of the neurons, 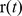, as
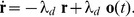
(3)


Our second assumption is that the network minimizes the distance between 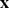 and 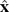 by optimizing over the spike times 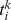, and not by changing the *fixed* output weight matrix 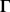. This approach differs from the “liquid computing” approach in which recurrent networks have fixed, random connectivities while the decoding weights are learnt [27]. In our case, the decoding weights are chosen a-priori. In order to track the temporal evolution of 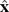 as closely as possible, the network minimizes the cumulative mean-squared error between the variable and its estimate, while limiting the cost in spiking. Thus, it minimizes the following cost function,
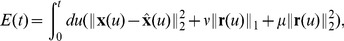
(4)where 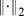 denotes the Eucledian distance (or L2 norm), and 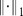 the Manhattan distance (or L1 norm), which here is simply the sum over all firing rates, i.e., 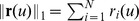. Parameters 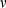 and 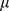 control the cost-accuracy tradeoff. The linear cost term, controlled by 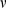, forces the network to perform the task with as few spikes as possible, whereas the quadratic cost term, controlled by 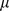, forces the network to distribute spikes more equally among neurons, as explained in Material and Methods.

### Network dynamics

To derive the network dynamics, we assume that the firing mechanism of the neurons performs a greedy minimization of the cost function 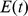. More specifically, neuron 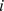 fires a spike whenever this results in a decrease of 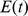, i.e., whenever 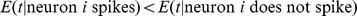. As explained in Material and Methods, this prescription gives rise to the firing rule
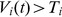
(5)with
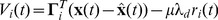
(6)

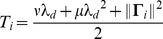
(7)Since 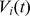 is a time-varying variable, whereas 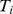 is a constant, we identify the former with the 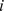-th neuron's membrane potential 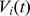, and the latter with its firing threshold 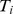.

In the limit 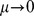, the membrane potential of the 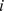-th neuron can be understood as the projection of the prediction error 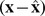 onto the output kernel 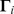. Whenever this projected prediction error exceeds a threshold, a new spike is fired, ensuring that 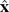 precisely tracks 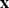. For finite 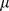, the membrane voltage measures a penalized prediction error. If the neuron is already firing at a high rate 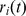, only a correspondingly larger error will be able to exceed the threshold and lead to a spike.

To connect this firing rule with the desired network dynamics, Eqn. (1), we take the derivative of each neuron's membrane potential, Eqn. (6), and consider the limit of large networks (see Material and Methods) to obtain the differential equation
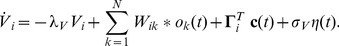
(8)where 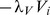 is a leak term, 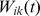 is a weight matrix of connectivity filters, explained below, and 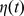 corresponds to a white “background noise” with unit-variance. The leak-term does not strictly follow from the derivation, but has been included for biological realism. A similar rationale holds for the noise term which we add to capture unavoidable sources of stochasticity in biological neurons due to channel noise, background synaptic input, etc. The differential equation then corresponds to a standard LIF neuron with leak term 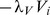, external, feedforward synaptic inputs 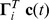, recurrent synaptic inputs mediated through the weight matrix 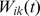, and a firing threshold 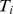, as specified in Eqn. (7).

The weight matrix of connectivity filters is defined as
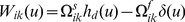
(9)and contains both “fast” and “slow” lateral connections, given by the matrices
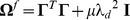
(10)

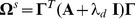
(11)where 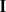 corresponds to the identity matrix. Accordingly, the connectivity of the network is entirely derived from the output weight matrix 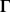, the desired dynamics 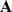, and the penalty parameter 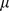. Note that the diagonal elements of 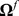 implement a reset in membrane potential after each spike by 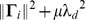. With this self-reset, individual neurons become formally equivalent to LIF neurons. Whereas the linear penalty, 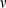, influences only the thresholds of the LIF neurons, the quadratic penalty, 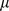, influences both the thresholds, resets, and dynamics of the individual neurons, through its impact on the diagonal elements of the connectivity matrix.

Slow and fast lateral connections have typically opposite effects on postsynaptic neurons, and thereby different roles to play. The fast connections, or off-diagonal elements of the matrix 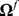, implement a competition among neurons with similar selectivity. If neuron 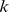 fires, the corresponding decreases in prediction errors (
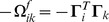
) are conveyed to all other neurons 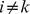. Neurons with similar kernels (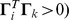 inhibit each other, while neurons with opposite kernels (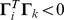) excite each other. This is schematized by the blue and red connections in Fig. 1 A.

In contrast, the slow connections, 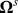, implement a cooperation among neurons with similar selectivity. These connections predict the future trajectory of 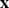 (term “
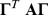
”) but also compensate for the loss of information due to the decoder leak (term “
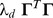
”). For example, when the variable 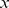 is static (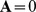, 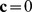), these connections maintain persistent activity in the network, preventing the variable 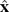 from decaying back to zero (see below). Note that when the internal dynamics of 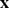 change on a slower time scale than the decoder (i.e. 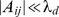), and if we neglect the cost term 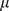, slow and fast connections have the same profile, (i.e. 
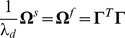
), but opposite signs.

The combined effect of fast and slow connections yields the effective PSPs in our network, 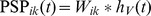, with 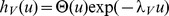, which can be obtained by integrating Eqn. (8) for a single spike. Two example PSPs are shown in Fig. 1 B. We note that our network model may contain neurons that both inhibit and excite different targets, depending on the kernel sign, a violation of Dale's law. This problem can be addressed by creating separate cost functions for excitatory and inhibitory neurons, as laid out in full detail in Text S1. Here, we simply interpret the resulting connectivity as the effective or functional connectivity of a network, akin to the types of connectivities arising in generalized linear models (GLMs) of neural networks [28].

### Scaling and physical units

We now briefly consider how the above equations can be mapped onto realistic physical units. This consideration has the additional benefit that it clarifies how the network parameters scale with the number of neurons 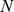 (see also Material and Methods). In order to express the network dynamics in biophysically relevant units, the membrane potential 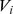, Eqn. (6), and threshold 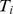, Eqn. (7), have to be rescaled accordingly. We can obtain proper membrane potential units in mV if we apply the simple transformations 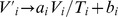 and 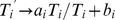. In turn, we obtain the modified equations
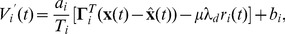
(12)

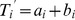
(13)and the modified dynamics
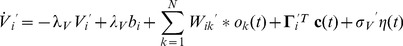
(14)with a resting potential of 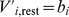. Note that both the feedforward and recurrent connectivities change in this case. Specifically, we obtain 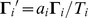 and 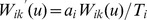, and a similar expression for the noise, 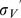. In turn, we can freely choose 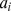 and 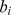 to find realistic units. For instance, we can fix the threshold at 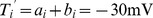, and the reset potential at 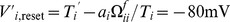, which uniquely determines both 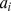 and 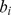 for each neuron. In the absence of linear costs (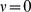), the reset potential becomes simply 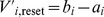.

When we increase the network size 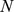 while keeping the average firing rates and the read-out constant, we need to change the decoding kernels. Specifically, the decoding kernels need to scale with 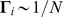. If we assume that the relative importance of the cost terms is held fixed for each neuron, then the original threshold 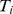 scales with 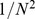, and the original connectivities 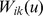 similarly scale with 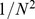, compare Eqns. (9–11). As a consequence, the rescaled synaptic weights, 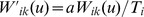 do not scale in size when the network becomes larger or smaller. When considering the summation over the different input spike trains, we therefore see that all synaptic inputs into the network scale with 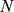: the feedforward inputs, the slow recurrent input, and the fast recurrent inputs (the latter two are both contained in the matrix 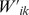). The equal scaling of all inputs maintains the detailed balance of excitation and inhibition in the network.

An instructive case is given if we neglect the cost terms for a moment (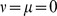) in which case we obtain the following (rescaled) feedforward weights and connectivities:
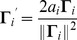
(15)

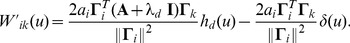
(16)Accordingly, the strength of the lateral connections is independent of the kernel norm. In contrast, the strength of the feed-forward connections scales with the inverse of the kernel norm. Since smaller kernels provide a more precise representation, the precision of the rescaled network, and its firing rates, are controlled entirely by its input gain.

### Sensory integration and sensory tracking

Once the dynamics and the decoders are chosen, Eqn. (1) and Eqn. (2), the only free parameters of the model are 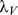, 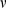, 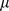, and 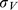. The model presented previously can in principle implement any linear dynamical system. We will first illustrate the approach with the simplest linear dynamical system possible, a leaky integration of noisy sensory inputs 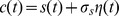 where 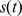 can be interpreted as the sensory stimulus while 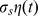 represents shared sensory noise. The corresponding dynamical system, Eqn. (1), is then given by
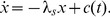
(17)The integrated sensory signal 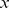 is a scalar (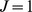) and 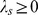 represents the leak of the sensory integrator.

For a completely homogeneous network, in which the output kernels 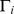 of all neurons are the same, we can solve the equations analytically which is shown in Text S1. A slightly more interesting case is shown in Fig. 1 C,D, which illustrate network dynamics for two different choices of 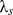. Here we used 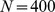 neurons, half of them with positive kernels (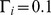), and the other half with negative kernels (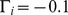). Neurons with positive kernels fire when variable 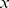 is positive or increases, while neurons with negative kernels fire when the variable is negative or decreases. Moreover, we set the cost terms 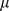 and 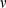 at small values, ensuring that our objective function 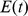 is dominated by the estimation error, compare Eqn. (4). As a consequence, the estimate 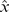 closely tracks the true variable 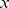. Albeit small, the cost terms are crucial for generating biologically realistic spike trains. Without them, a single neuron may for example fire at extremely high rates while all others stay completely silent. The regularizing influence of the cost terms is described in more detail in Text S1.

For 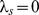, the network is a perfect integrator of a noisy sensory signal. The neural activities resemble the firing rates of LIP neurons that integrate sensory information during a slow motion-discrimination task [1]. In the absence of sensory stimulation, the network sustains a constant firing rate (Fig. 1 C after 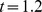
sec), similar to line attractor networks [29]–[31]. In fact, as long as the dynamics of the system are slower than the decoder (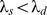), the instantaneous firing rates approximate a (leaky) integration of the sensory signals. On the other hand, if the system varies faster than the decoder (i.e. 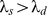), then neural firing rates track the sensory signal, and model neurons have transient responses at the start or end of sensory stimulation, followed by a decay to a lower sustained rate (Fig. 1 D). These responses are similar to the adaptive and transient responses observed in most sensory areas.

The overall effect of the lateral connections depends on the relative time scales of the variable 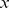 and the decoder 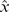 (Fig. 1 B). For neurons with similar selectivity (or equal read-out kernels, 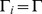), the postsynaptic potentials are given by (assuming 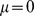),
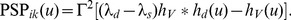
(18)For neurons with opposite read-out kernels, we obtain just a sign reversal. When (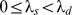), the interplay of fast inhibition with slower excitation results in a bi-phasic interaction between neurons of similar selectivity (Fig. 1 B, left). Moreover, the network activity persists after the disappearance of the stimulus. In the extreme case of the perfect integrator (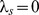), the temporal integral of this PSP is exactly zero, which explains how the mean network activity can remain perfectly stable (neither increase nor decrease) in the absence of any sensory stimulation. In contrast, lateral interactions are entirely inhibitory when the network tracks the stimulus on a faster time scale than the decoder (i.e. 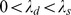, Fig. 1 B, right). The dominance of lateral inhibition explains the transient nature of the network responses and the absence of persistent activity.

**Figure 2:**
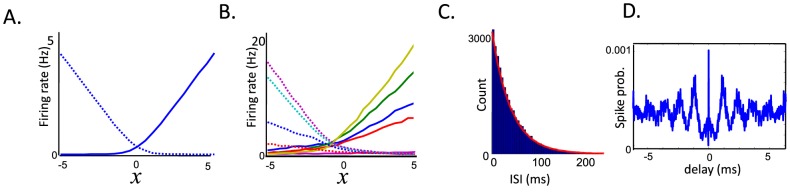
Response properties of the sensory integrator. (A) Tuning curves to variable 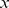 for the network with uniform kernels. Plain line: 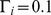. Dashed line: 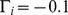. Parameters are as in Fig. 1 C. Tuning curves were obtained by providing a noiseless (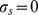) sensory input 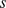 of various strength during the first 250 ms, then measuring sustained firing in the absence of inputs during the next 1000 ms. The response shown is averaged over 500 trials. (B) Example tuning curves for the inhomogeneous network (Plain lines: all components of 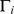 positive. Dashed lines: all elements of 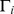 negative). Parameters are 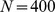, 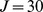, 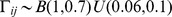 for 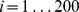, 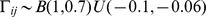 for 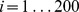, 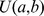 is a uniform distribution within 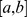, 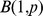 is a binomial distribution, 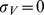, 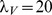
Hz, 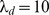
Hz, 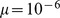, 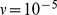, 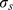 = 0, based on a simulation with the Euler method and time step 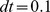
msec. (C) Inter-spike interval distribution for a typical unit (inhomogeneous network with 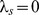). ISI distribution is measured during “persistent activity” in the absence of sensory stimulation (firing rate is constant at 5 Hz). Red lines show the prediction from a Poisson process with the same rate. (D) Mean cross-correlogram for a pair of units with the same kernel sign (inhomogeneous network). Probability of a spike in unit 1 is plotted at different delays from a spike in unit 2.

Other response properties of the model units are illustrated in Fig. 2. We define the tuning curves of the neurons as the mean spike count in response to a 1 s presentation of a constant stimulus 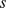. Firing rates monotonically increase (for positive kernels) or decrease (for negative kernels) as a function of 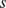 and are rectified at zero, resulting in rectified linear tuning curves (Fig. 2 A). Since all neurons have identical kernels (i.e. all 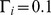 or 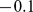), neurons with the same kernel signs have identical tuning curves. However, such a homogeneous network is rather implausible since it assumes all-to-all lateral connectivity with identical weights, so that all units in the network receive exactly the same synaptic input and have the same membrane potential.

**Figure 3:**
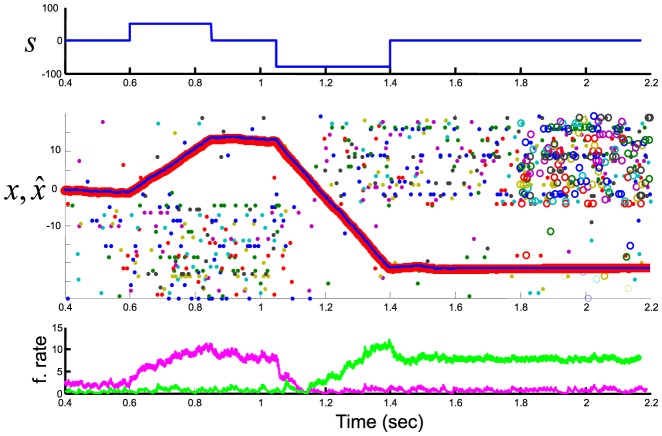
Response of the inhomogeneous integrator network. Same format as in Fig. 1 C. The network is entirely deterministic (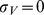). Top panel: sensory input (blue line). Before 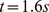, the sensory signal 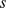 is corrupted by sensory noise with variance 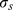. Sensory input and sensory noise stop after 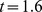
s, at which point the network is entirely driven by its own internal and deterministic dynamics. The network is simulated twice using exactly the same initial conditions and input 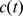. Up to 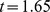
s, the two simulations give exactly the same spike train as represented by the dots (deterministic network with identical inputs). At 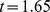
s, a small perturbation is introduced in the second simulation (a single spike is delayed by 1 ms). The subsequent spike trains are completely re-shuffled by the network dynamics (First simulation: dots. Second simulation: circles). Simulation parameters are 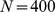, 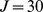, 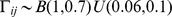 for 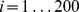, 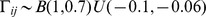 for 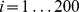, 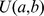 is a uniform distribution within 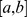, 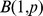 is a binomial distribution, 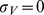, 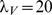
Hz, 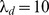
Hz, 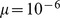, 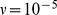, 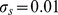, based on a simulation with the Euler method and time step 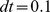
msec. Bottom panel shows the mean instantaneous firing rate for the population of neurons with positive kernels (magenta) and negative kernels (green) measured in an exponential time window with width 100 ms.

To move to more realistic and heterogeneous networks, we can choose randomized decoding kernels 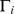. Even then, however, the connectivity matrix 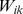 is strongly constrained. For negligible costs, 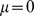, the weight matrix has rank one (since 
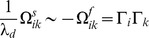
). A lot more flexibility can be introduced in the network connections by simultaneously tracking 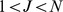 variables with identical dynamics and identical control 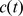, rather than a single scalar variable. Thus the variable 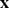 and the kernels 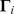 have 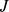 dimensions and 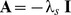. We then define the actual network output, 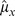, as the mean of those 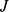 variables (simply obtained by summing all network outputs). The network estimation error, 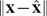, is an upper bound on 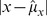, ensuring similar performance as before (see Fig. 3). Importantly, we can choose the output kernels 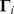 to fit connectivity constraints imposed by biology. For instance, the output kernels can be made random and sparse (i.e. with many zero elements), resulting in random and sparse (but symmetrical) connection matrices. In such a network, the tuning curves are still rectified-linear, but have different gains for different neurons (Fig. 2 B).

Output spike trains of both homogeneous and inhomogeneous networks are asynchronous and highly variable from trial to trial (see raster plots in Fig. 1 C,D and Fig. 2). Fano factors (measured during periods of constant firing rates), CV1, and CV2, were all found to be narrowly distributed around one. The interspike interval (ISI) distribution was close to exponential (Fig. 2 C). Moreover, noise correlations between neurons are extremely small and do not exceed 0.001 (noise correlations are defined as the cross-correlation coefficient of spike count in a time window of 1 s in response to a constant variable 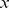). Finally, analysis of auto and cross-correlograms reveals the presence of high-frequency oscillations at the level of the population (Fig. 2 D). These high frequency oscillations are not visible on Fig. 2 C since the size of the bin (5 ms) is larger than the period of the oscillations (1 ms). Note that if we add a realistic amount of jitter noise (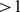
ms) to spike times, these high frequency oscillations disappear without affecting the response properties of the network.

**Figure 4:**
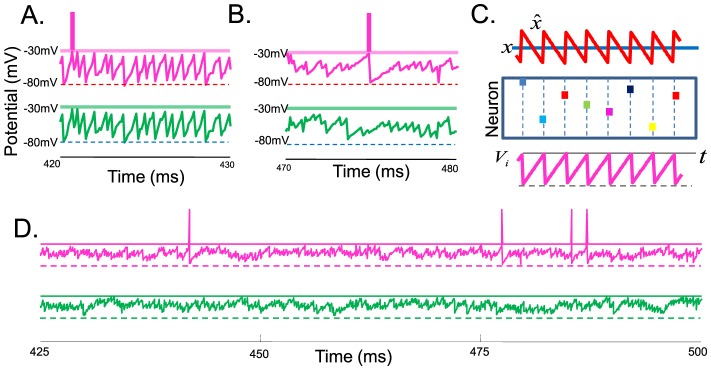
Membrane potential profiles for the integrator networks. (A) Homogeneous network. Example profiles for two neurons with identical kernels. Vertical line represents a spike in the red unit, plain horizontal line represents the firing threshold, and dashed horizontal line the reset potential. Values are interpreted in mV after rescaling the membrane potential (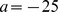
mV and 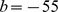
mV). These profiles are taken from the simulation shown in Fig. 1 C. (B) Inhomogeneous network. Membrane potential profiles for two neurons with strongly correlated kernels (i.e. large 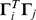) and no synaptic background noise (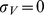). These profiles are taken from the simulation shown in Fig. 3. (C) Schema explaining the distribution of spikes across neurons in a homogeneous network (see text). (D) Same two units as in (B) shown for a longer period of time.

In contrast to the output spike trains, the membrane potentials of different neurons are highly correlated, since neurons with similar kernels (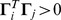) receive highly correlated feed-forward and lateral inputs (Fig. 4 A,B). In the homogeneous networks without quadratic cost (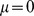), these inputs are even identical, resulting in membrane potentials that only differ by the background noise (Fig. 4 A). Despite these strong correlations of the membrane potentials, the neurons fire rarely and asynchronously. Fig. 4 C illustrates why this is the case: let us consider a population of neurons with identical output kernels 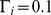, maintaining an estimate of a constant positive 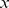 (top panel, blue line). Due to the decoder leak 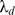, the network needs to fire periodically in order to maintain its estimate 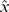 at the level of 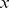 (top panel, red line). However, the exact order at which the different neurons fire does not matter, since they all contribute equally. The period between two spikes can be called an “integration cycle”. Within one integration cycle, the prediction errors and thus the membrane potentials, 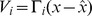, rise for all neurons (bottom panel, red line). Since all kernels are identical, however, all neurons compute the same prediction error and will reach their firing thresholds at approximately the same time. Only chance (in this case, the background noise 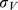) will decide which neuron reaches threshold first. This first neuron is the only one firing in this integration cycle (middle panel, colored bars) since it immediately inhibits itself and all other neurons. This starts a new integration cycle. As a result of this mechanism, while the population of neurons fire at regular intervals (hence the high frequency oscillations in Fig. 2 D) only one neuron fires in each cycle, and its identity is essentially random. The resulting variability has no impact on the network estimate, since all spike orders give the same output 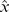. In the presence of a quadratic cost (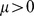), neurons that did not fire recently have a higher probability of reaching threshold first (their membrane potential is not penalized by 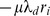). When the cost term is large compared to the background noise (i.e. when 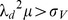, which is not the case in the example provided here), this tends to regularize the output spike trains and leads to 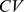
s smaller than 1. However, this regularization is not observed in inhomogeneous networks.

The inhomogeneous network behaves similarly, except that all neurons do not receive the same inputs and do not reach threshold at the same time (Fig. 4 B). In this case, we can even dispense of the background noise (i.e. 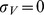) since fluctuations due to past network activity will result in a different neuron reaching threshold first in each cycle. The individual ups and downs caused by the synaptic inputs from other neurons will nonetheless appear like random noise when observing a single neuron (Fig. 4 B,D). Furthermore, even in this deterministic regime, the spike trains exhibit Poisson statistics. In fact, changing the timing of a single spike results in a total reordering of later spikes, suggesting that the network is chaotic (as illustrated in Fig. 3).

### 2D arm controller

We now apply this approach to the tracking of a 2D point-mass arm based on an efferent motor command. The dynamical variable has 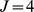 dimensions corresponding to the arm positions 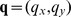 and the arm velocities 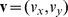. The arm dynamics are determined by elementary physics, so that
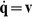
(19)

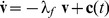
(20)where 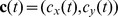 is a 2D (control) force exerted onto the arm, and 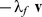 captures possible friction forces.

**Figure 5:**
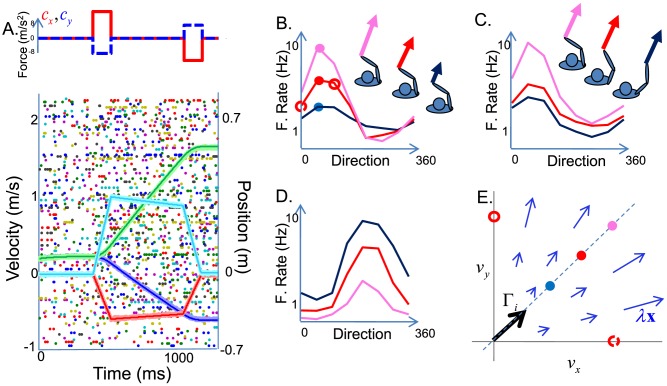
Spike-based implementation of a 2-D arm forward model. (A) Network response for a reaching arm movement. Top panel: Control variables (force exerted on the arm in 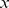 and 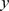 axis). Bottom panel: raster plot for a sub-population of 140 neurons. Thin lines: Real arm state 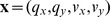; Thick lines: network estimate 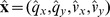. Thin and thick lines are perfectly superposed. Blue and green: positions 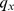 and 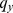. Red and cyan: velocities 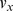 and 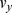. (B) Tuning curve to direction for an example unit. Blue, Red and Magenta correspond respectively to arm speed of 0.2, 0.5, and 1 m/s, as represented by the inlaid schemata. (C) Tuning curves to direction (same neuron as in B) tested at 3 different arm starting position. Blue, Red and magenta correspond to arm position 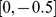, 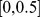 and 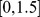. (D) Direction tuning at 3 different arm positions for another example unit (same legend as C). (E) Schema explaining the tuning properties of model units. Dots in panels B and E represents the same arm state. Parameters in A–D: 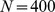, 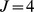, 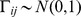, normalization constraint 
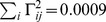
, 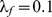, 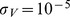, 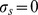, 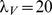
Hz, 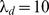
Hz, 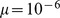, 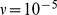, 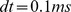.

To simulate this system, we studied an arm moving from a central position towards different equidistant targets. This reaching out arm movement was obtained by “push-pull” control forces providing strong acceleration at the beginning of the movement, and fast deceleration at the end of the movement (Fig. 5 A, top panel). As previously, the network predicts the trajectory of the arm perfectly based on the forces exerted on it (Fig. 5 A, bottom panel; we again use relatively small cost terms 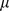 and 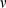). The resulting spike trains are asynchronous, decorrelated, and Poisson-like, with unpredictable spike times (rasters in Fig. 5 A; Fano factor and CVs close to 1). The membrane potential of neurons with similar kernels are correlated while output spike trains are asynchronous and decorrelated. The effective postsynaptic potentials have biphasic shapes reflecting the integrative nature of the network for small friction forces (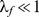).

To measure tuning curves in this “center out” reaching task, we varied the speed and direction of the movement, as well as the starting position of the arm. Neural activity was defined as the mean spike count measured during movement. As illustrated in Fig. 5 B,C,D, instantaneous firing rates are modulated by arm position, velocity and force. We found that tuning curves to arm position are rectified linear, with varying thresholds and slopes (as in Fig. 2 B). Such linear-rectified gain curves with posture have been reported in premotor and motor cortical areas [32], [33]. In contrast, tuning curves to circular symmetric variables such as movement direction or arm angle are bell-shaped (Fig. 5 B,C,D). In addition, direction tuning curves are gain modulated by arm speed, such that responses are stronger for larger speed when the arm moves in the preferred direction, and weaker when the arm moves in the anti-preferred direction (Fig. 5 B). Finally, arm positions have both an additive and a gain modulating effect on the tuning curve, and these modulation can be monotonically increasing (Fig. 5 C) or decreasing (Fig. 5 D) with arm position.

These observations have a simple geometric explanation, schematized in Fig. 5 E for the velocity space, 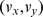. A neuron is maximally active (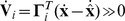; assuming 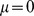) when its kernel (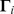, thick vector in Fig. 5 E) points in the direction of the derivative of the prediction error, 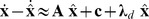. Since the decoder leak is faster than the arm dynamics, this error mostly points in the direction opposite to the leak, 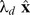 (thin vectors). Within the velocity space, the kernel thus defines the neuron's preferred movement direction (dashed line and filled circles). The neurons is less often recruited when the arm moves away from the kernel's direction (empty circles), resulting in a bell-shaped tuning curve. Finally, since the vector 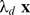 gets larger at larger speeds, more spikes are required to track the arm state resulting in a linear tuning to movement speed. The same reasoning applies for the position space 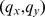. These predictions are independent of the choice of kernels and are in direct agreement with experimental data from the pre-motor and motor cortices [32], [33].

### Differentation and oscillation with heterogeneous networks

**Figure 6:**
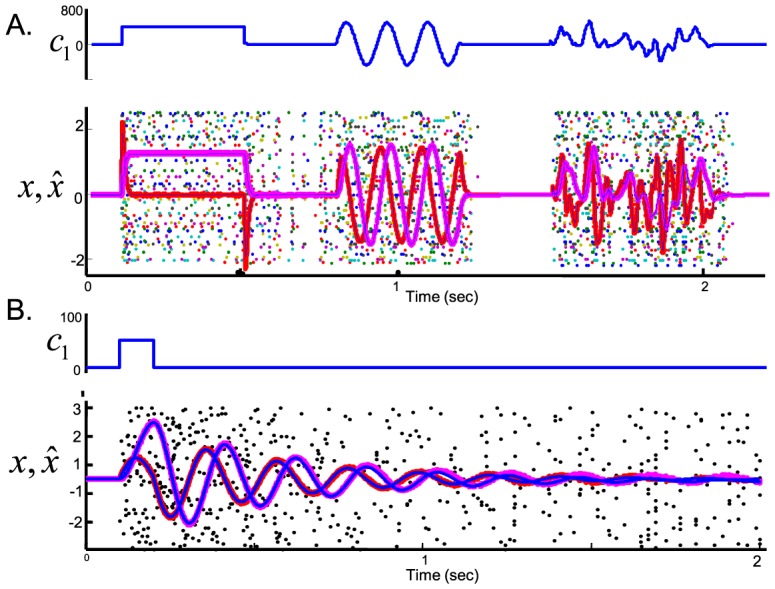
Other example networks, same format as Fig. 3. (A) Neural implementation of a “leaky differentiator”. The network tracks two dynamical variables with a state transition matrix 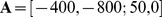. Top panel: command variable 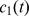. (Note that 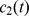 is zero.) Bottom panel: network response and estimates. Thick red and purple lines: Network estimates 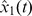 and 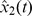. Thin blue lines: Variables 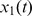 and 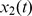. The variables and network estimates perfectly track each other, making the thin blue lines hard to see. Overlaid dots represent the corresponding output spike trains, with a different color for each neuron. (B) Neural implementation of a damped harmonic oscillator. The network tracks two dynamical variables with 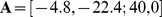. Format as in A. Simulation parameters for A and B: 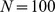 2-D vectors 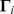 were generated by drawing each coordinate from a normal distribution and normalizing the vectors to a constant norm, so that 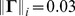. Other parameters were 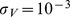, 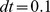
ms, 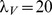
Hz, 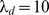
Hz, 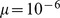, 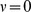. Dots represent spike trains, one line per neuron, shown in black to improve visibility.

We chose to present a sensory integrator and an arm controller for their biological relevance and simplicity. However, any linear dynamical system can be implemented within our framework, and our networks are not limited to performing integration. To illustrate the generality of the approach, we applied the framework to two additional examples. In Fig. 6 A, we simulated a “leaky differentiator” with a transition matrix 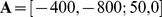. This system of differential equations is designed so that the variable 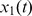 approximates a temporal derivative of a command signal 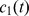. The command signal, 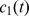, is shown in the top panel of Fig. 6 A; the input signal 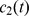 is zero. We used 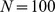 neurons with kernels drawn from a normal distribution, and then normalized to a constant norm of 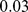. As in the other examples, the firing statistics are close to Poisson, with a 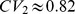.

In Fig. 6 B, we simulated a network that implements a damped harmonic oscillator. Here we chose a transition matrix 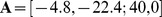. The oscillator is initially kicked out of its resting state through a force given by the command signal 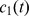, as plotted on the top panel. The input signal 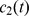 is zero. We used 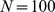 neurons with kernels drawn from a normal distribution, and normalized to a constant norm of 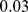. The network tracks the position 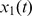 and speed 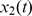 of the damped oscillator until position and speed are too close to zero to allow a reliable approximation. The firing statistics of single units are again Poisson-like, with 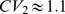.

Note that in these two examples, the dynamics implemented by the network are faster than the decoder's time scale 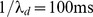. Accordingly, our networks can track changes faster than the time scale of the decoder. This speed-independence relies on a simple scheme: Spikes from neurons with positive kernel weight, 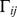, represent instantaneous increases in 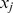, whereas spikes from neurons with negative kernel weight 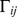 represent instantaneous decreases in 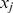. Even if the inter-spike interval is much shorter that 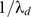, the decoder can therefore still provide an efficient staircase approximation for 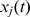. In conclusion, the temporal accuracy of these networks is not limited by 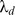, but by 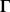.

## Discussion

We have proposed a method for embedding any linear dynamical system in a recurrent network of LIF neurons. The network connectivity and spike generation are entirely derived from a single loss function which seeks to optimally place spikes so that the relevant information can be extracted by postsynaptic integration. Accordingly, the network structure follows exclusively from functional principles, and no extensive parameter searches are required. This approach implies in particular that neurons share information in a smart way, yet fire almost randomly at the level of the single cell.

We also included a cost term in the error function, Eqn. (4). Due to this cost term, the network finds a solution minimizing the metabolic cost associated with high spike counts. Both linear and quadratic cost terms regularize the firing rate and make the network robust against artefacts such as high firing rates that may be caused by the greedy spiking mechanism (see Text S1). Further generalizations or modifications of these predictive coding principles may eventually help to explain other biophysical or electrophysiological phenomena of the brain.

### Relation to other approaches

Our current work both generalizes and modifies our earlier work in which we applied the principle of predictive coding with spikes to a Bayesian inference problem [20]. This model tracked a log-probability distribution and implemented a non-linear drift-diffusion model, rather than a generic linear differential equation. In addition, we here introduced cost terms which provided us with greater flexibility in regulating and controlling the dynamics of the spiking networks.

A quite general framework for designing networks of neurons that implement arbitrary dynamical systems has previously been described in the “neuro-engineering” approach [30]. This approach relies on linearly combining the non-linear rate transfer function of LIF neurons. In its essence, the method is therefore based on firing rates, and makes few predictions about the spiking statistics of cortical neurons. A recently developed model, the “ReFiRe network” [34] provides a recipe for designing networks maintaining stable memories, and shares some of the features of our networks. Just as the neuro-engineering framework, however, its design is essentially based on firing rates.

**Figure 7:**
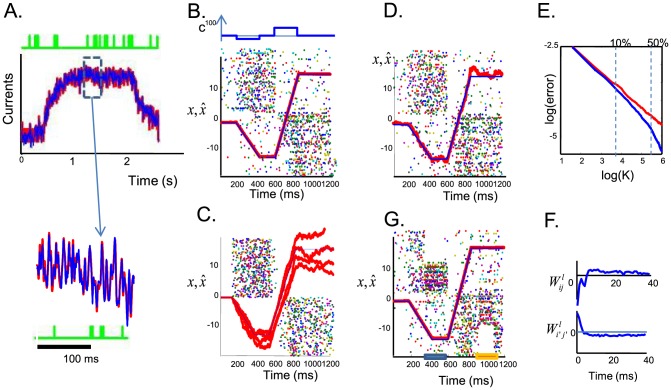
Distinguishing spiking codes from Poisson rate codes. (A) Example profile of total excitatory current (red) and inhibitory current (blue) in a single unit on two different time scales (time scale of the stimulus 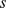 and time scale of an inter-spike interval). Currents were convolved with a 2 ms exponential time window. (B) Response of the homogeneous integrator network (same parameters as in Fig. 1 C). The input 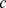 is shown in the top panel. (C) Spike trains (dots), true state 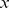 (blue), and estimate 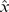 (red) for a rate model with the same slow connections and input as in B. Fast connections were removed and the greedy spiking rule was replaced by a random draw from an equivalent instantaneous firing rate. Four different trials are shown (four thick red lines) to illustrate the variability in the rate model's estimate. (D) Spike trains (dots), state 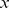 (blue) and estimate 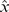 (red) when each spike train is recorded from a different trial of the network shown in (B). (E) Estimation error, 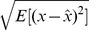, as a function of the number of recorded neurons, 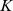, for a spiking network with 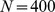 neurons. For the blue line, all 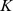 neurons were recorded simultaneously, for the red line, each neuron is recorded in a different trial (red). The red line follows perfectly the prediction for 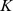 independent Poisson processes. Data are from an homogeneous integrator network with 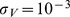 and 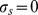, other parameters as in Fig. 1 C. (F) Effective connectivity filters of two randomly chosen pairs in the network, as measured through a GLM analysis. (G) Consequence of suddenly inactivating half of the active neurons for the network shown in B. Blue bar: unit 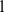 to 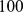 inactivated (membrane potential set to 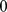). Orange bar: units 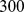 to 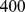 inactivated. Other parameters as in Fig. 2 B–D,F.

Here we have designed a network based on the principle of predictive coding with spikes. Even though indistinguishable from older models on the single cell level, our work is different in several important respects. A first major difference of our approach is that it predicts a detailed balance between excitation and inhibition, rather than imposing it upfront. This balance follows from the attempt of the network to minimize the loss function, Eqn. (4), which in turn implies that the membrane potential of neurons represents a prediction error and that neurons spike only when this prediction error exceeds a certain value—a form of predictive coding. Any increase in excitation causes an increase in prediction error, immediately compensated by an increase in inhibition to bring down the prediction error (and vice versa). This interplay causes a tight temporal correlation between excitation and inhibition at the time scale of the stimulus but also at a much finer time scale, within a single ISI (Fig. 7 A). Note that this balance only holds when considering all inputs. In the leaky integrator, for instance, all lateral connections are inhibitory (Fig. 1 B, right panel). However, the network is still globally balanced when taking into account the contribution from the feedforward connections. Such a tight balance between excitation and inhibition has been observed at several levels of cortical processing [22]–[26].

Accordingly, spike trains in our network usually resemble independent Poisson processes, with rates tuned to the variable 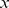. We note that spike trains can also be more regular if the networks are smaller and the noise is not too large. A simple example is a network composed of a single neuron (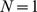), for which we provide an analytical solution in Text S1. Such a LIF neuron responds to a constant positive input with a perfectly regular spike train. In practice, regular firing is observed when only a few neurons are simultaneously co-active (e.g. for networks composed of less than 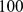 neurons). Firing becomes irregular when many neurons are co-active (e.g. for networks of several hundreds of neurons or more). Increasing synaptic background noise tends to make firing less regular, while increasing the quadratic metabolic costs makes firing more regular. However, for large networks, these effects are small and remain within the range of Fano-factors or CVs observed in cortex. The amount of regularity has no impact on the network performance.

Despite the variability observed in large networks, one cannot replace or approximate one of our spiking networks with an equivalent rate model composed of Poisson spike generators, a second major difference to other network models. This point is illustrated in Fig. 7 B,C for the homogeneous integrator model, where we removed the fast connections in the network and replaced the integrate-and-fire dynamics by 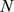 independent Poisson processes (see Material and Methods). The performance of the resulting Poisson generator network is strongly degraded, even though it has the same instantaneous firing rates and slow connections as the LIF network.

We can quantify the benefit of using a deterministic firing rule compared to stochastic rate units by considering how the estimation error scales with the network size. The integrator model tracks the dynamical variable 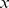 with a precision defined by the size of a kernel 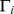. Estimation errors larger than 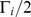 are immediately corrected by a spike. As the network size 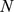 increases, maintaining the same firing rates in single units requires that the kernels, and thus, the estimation error, scale with 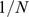 (see Material and Methods). In contrast, the error made when averaging over a population of independent Poisson neurons diminishes with 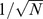. Intuitively, the predictive coding network achieves higher reliability because its neurons communicate shared information with each other via the fast synapses, whereas the independent Poisson neurons do not. The communicated information actively anti-correlates all spike trains, which, for networks composed of more than a dozen neurons, will be indistinguishable from the active decorrelation of pairwise spike trains that has recently been observed in vivo [35]. Therefore, the precision of the neural code cannot be interpolated from single-cell recordings in our network, and combining spike trains recorded in different trials results in a strong degradation of the estimate (Fig. 7 D).

A third major difference between our network model and those proposed previously concerns the scaling of the network connectivity. Most previous approaches assumed sparse networks and weak connectivity in which the probability of connections (and/or connection strengths) scales as 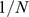 or 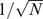. This weak connectivity leads to uncorrelated excitation and inhibition and thus neurons driven by random fluctuations in their input [15], [36]. For comparison, the connectivity in our network is finite (once the membrane have been rescaled by the kernel norm to occupy a fixed range of voltage). Our approach is therefore reminiscent of a recent model with finite connection probability [17]. As in our model, stronger connectivity leads to correlation between excitation and inhibition but uncorrelated spike trains. The strong network connectivity in turn swamps the membrane potential of each neuron with currents. The excitatory and inhibitory currents driving the neural response grow linearly with the number of neurons, 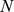, and are thus much larger than the membrane potential (prediction error) 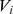, which is bounded by the (fixed) threshold. In turn, the leak currents 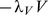 become negligible in large networks. For example, the integrator network in Fig. 1 C has 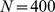 neurons and can maintain information for 100 s (it takes 100 seconds for the network activity to decay by half) despite the fact that the membrane time constant (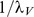) is only 0.1 s. Thus, according to our model, spiking neurons can fire persistently and thereby maintain information because their leaks are dwarfed by the currents they receive from recurrent connections.

### Network limitations

There are several non-trivial circumstances under which our network models may fail. First, we notice that the spiking rule that we derive amounts to a greedy optimization of the loss function. Future costs are not taken into account. This may cause problems in real neurons which can only communicate with time delays, but it may also cause problems when neurons have opposing kernels. For instance, two neurons with opposing kernels may become engaged in rapidly firing volleys of spikes, each trying in fast succession to decrease the error introduced by the previous spike from the other neuron (see Text S1), a problem that we call the “ping-pong” effect. This effect can become a serious problem if the network dynamics is corrupted by strongly correlated noise, which may occur in the presence of synaptic failures. However, it is usually possible to diminish or eliminate this effect by increasing the spike count cost (see Text S1).

Second, the leak term we introduced in the voltage equation provides only an approximation to the actual voltage equation (see Material and Methods). Specifically, the term we approximate is 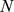 times smaller than the other terms in the membrane potential dynamics. In practice, we can therefore always increase the network size to reach an acceptable level of performance. For a given network size, however, the approximation may break down when 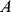 becomes too large or when both 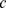 and 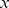 are too small (of order 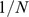).

Third, the speed at which the linear dynamical system can evolve will be limited from a practical point of view, even in the limit of large networks. While the time scale of the decoder, 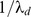 does not put any strict limitations on the speed (since spikes corresponding to positive and negative kernels can always provide an efficient stair-case approximation to any time-varying function), faster dynamics can only be obtained if the linear dynamical system compensates for the decoder filtering. This compensation or inversion process is a case of deconvolution, and bound to be severely limited in practice due to the noise inherent in all physical systems.

Finally, the network requires finely tuned lateral connections in order to balance excitation and inhibition (from feed-forward and lateral connections). In particular, the strength of the fast connections between two neurons corresponds to minus the correlation coefficient of their feed-forward connections (and thus, to their level of shared inputs). Whether such finely tuned motifs exist in biological networks is still an open question. We showed recently that fast lateral connections can be learnt using unsupervised Hebbian learning [37], suggesting that networks with the appropriate form of plasticity would be able to develop and maintain this tight balance. We note that the performance of the networks is quite sensitive to global perturbations of the balance between excitation and inhibition, an issue that we discuss in more detail in Text S1.

### Experimental predictions

The most crucial work left to the future will be to test the predictions derived from this theory, three of which are described here. First, one could test how the decoding error scales with the numbers of simultaneously recorded neurons. A single unit in the model network (considered in isolation) is in fact exactly as reliable as a Poisson spike generator with the same rate. As the number of simultaneously recorded neurons increases, the decoding error initially decreases as 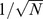, similar to a Poisson rate model. However, as the number of neurons reaches a certain threshold (10% for the network models simulated here), the error from the spiking network decreases faster than predicted for a Poisson rate model (Fig. 7 E). So far, single-unit recordings or multi-electrode recordings have only sampled from a very small subpart of the population, making it impossible to see this difference (and in turn, potentially leading to an under-estimation of the precision of the neural code). However, with newer techniques, such as dense multi-electrode arrays or optical imaging, as well as with focusing on smaller networks (such as the oculomotor integrator or insect systems), these model predictions are nowadays within experimental reach. We note that one has to carefully account for the effect of shared sensory noise (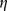) to see the predicted scaling effect. Shared noise (absent in Fig. 7 E) introduces correlations between neurons and results in a saturation of the error with 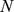. In our network, such a saturation would only be seen if there were limits to the sensory information available in the first place; saturation would not be seen as a consequence of neural noise or correlations (as proposed for example in [38], [39]).

Second, one could look at the global interaction between neurons of similar selectivity, for example by applying a GLM model to the data [28]. The model predicts that neurons involved in slow integration tasks or working memory tasks should inhibit and excite each other at different delays. In particular, neurons with similar selectivities should be (paradoxically) negatively correlated at short delays. Thus, applying GLM analysis even on a small sub-population can uncover the effective PSPs caused by the lateral connections and, indirectly, the dynamical equation implemented by the network. Fig. 7 F shows the GLM filters learnt from the inhomogeneous integrator network during working memory (i.e. sustained activity in the absence of sensory input). The analysis recovered the shape of the filters between neurons of similar kernels and opposite kernels, despite the fact that only 10 simultaneously recorded neurons (2.5% of the population) were used in this analysis.

Third, the spiking network is by essence self-correcting and will thus be resilient to lesions or many sudden perturbations (an exception being perturbations of the global balance of excitation and inhibition, see above). Equipping neural networks with such resilience or robustness has been a well-studied theoretical problem. For the specific example of the neural integrator, solutions range from constructing discrete attractor states [40], [41], [42], adding adaptation or learning mechanisms to a network [43], [44], or changing the nature of network feedback [45], [46]. In the case of the neural integrator, the robustness of our network could likely be interpreted as a case of derivative feedback [46].

While we know that biological neural networks are quite robust against partial lesions, their response to sudden, yet partial perturbations is less well known. For example, suddenly inactivating half of the active neurons in our sensory integrator increases the firing rates of the remaining neurons but has essentially no effect on the network performance (Fig. 7 G). This instantaneous increase in firing rates without performance loss generates a strong prediction for our network model, a prediction that distinguishes our network from previously proposed solutions to the robustness problem. Indeed, as long as the pool of available kernels remains sufficient to track 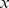, and as long as increased firing rates are not affected by saturation, inactivation will not affect the network's computation. This prediction could be tested using for example optogenetic methods.

## Materials and Methods

### Derivation of the spiking rule, Eqns. (5–7)

We here derive the network equations using compact matrix-vector notation. In Text S1, we also consider the special case of a homogeneous network and a single neuron, for which the derivations are simpler. We consider the error function, Eqn. (4), which is given by
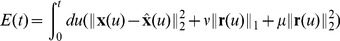
(21)The 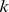-th neuron should spike at time 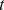 if
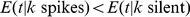
(22)A spike by the 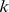-th neuron adds a single delta-function to its spike train. This additional spike enters the right-hand-side of the read-out equation, Eqn. (2). Integration of this extra delta-function amounts to adding a decaying exponential kernel, 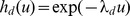 to the read-out. Hence, if neuron 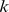 spikes at time 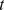, we have
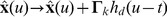
(23)

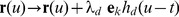
(24)where the latter equation describes the instantaneous change in firing rate due to the additional spike. Note that the standard Eucledian basis vector 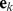 is a vector in which the 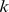-th element is one, and all others are zero.

Each spike influences the read-out several time intervals 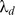 into the future. To see whether a spike leads to a decrease of the error function, we therefore need to look into the future (from time 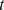 onwards). For a future time 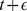 with 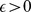, the spiking rule in Eqn. (22) translates into
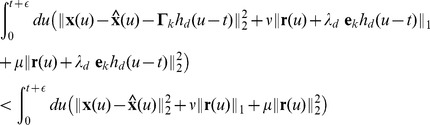
(25)We can expand the terms on the left-hand-side, and then eliminate identical terms on both sides. For that, we remind the reader that the relation 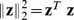 holds for the 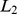 norm, whereas 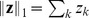 holds for the 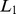 norm in our case, since all elements (firing rates) are positive by definition. Hence we obtain
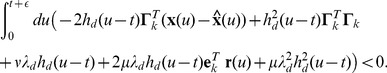
(26)We rearrange the inequality by moving all terms that depend on the dynamical variables 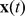, the estimates 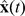, or the firing rates 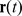 to the left, and all other terms to the right, and we then multiply both sides by minus one, to obtain
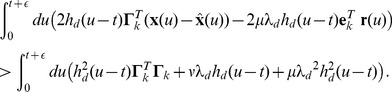
(27)Moving the kernels 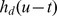 to the front of the integrals and noticing that 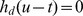 for 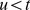, we obtain
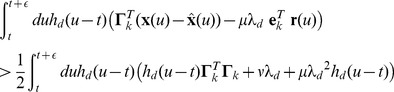
(28)The integral on the left-hand-side weights the influence of the spike, as given by the decaying exponential kernel, 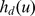, against the future development of the error signal, 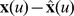, and firing rate 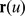. These future signals are unknown: while we may be able to extrapolate 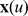, given its dynamical equation, we cannot safely extrapolate 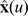 or 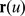, since this would require knowledge of all future spikes. We therefore choose a “greedy” approximation in which we only look a time 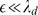 into the future. For the relevant times 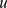, we can then approximate the integrands as constants so that (using 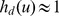 for 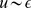)
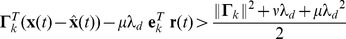
(29)which is our decision to spike, and corresponds exactly to Eqns. (5–7). We notice that the right-hand-side is a constant whereas the left-hand-side is a dynamical quantity which corresponds to the projection of the prediction error, 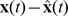, onto the output kernel of the 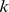-th neuron, 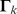, subtracted by a term depending on the firing rate of the 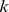-th neuron. Given this threshold rule, it seems only natural to identify the left-hand-side with the membrane voltage 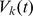 of the 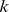-th neuron and the right-hand-side with its spiking threshold, 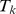, which is what we did in the main text.

### Derivation of the voltage equation, Eqn. (8)

If we write the voltage of all neurons as one long vector, 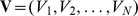, then we can write
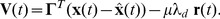
(30)We generally assume that there are more neurons than variables to represent so that 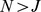. We also assume that the output kernel matrix, 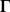, has rank 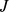, and that the dynamical variables are not degenerate or linearly dependent on each other. In this case, the left pseudo-inverse of 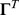 exists and is given by
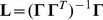
(31)so that 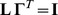. Note that 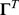 is an 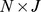-matrix, while 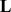 has size 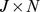. In turn, we can solve the voltage equation for 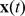 by multiplying with the pseudo-inverse from the left so that
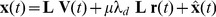
(32)Taking the derivative of the voltages, we obtain
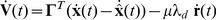
(33)Replacing 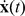, 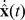, and 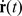 with their respective equations, Eqns. (1–3), we obtain
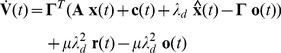
(34)In turn, we can replace 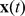 with Eqn. (32) to obtain
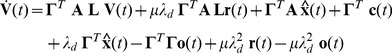
(35)Sorting some of the terms, and remembering that 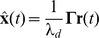, we obtain
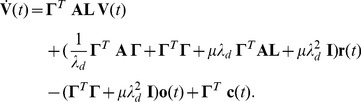
(36)


To evaluate the relative importance of the different terms, we consider the limit of large networks, i.e., the limit 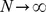. First, we impose that the average firing rates of individual neurons should remain constant in this limit. Second, we require that the read-out does not change. Given the scaling of the firing rates, and since 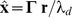, the output kernels must scale with 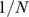. Accordingly, the pseudo-inverse 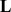 scales with 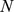. Finally, we need to choose how the cost terms 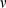 and 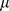, scale with respect to the read-out error. The linear and quadratic error terms 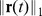 and 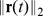 scale with 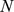. To avoid a contribution of the cost term increasing with network size, 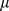 and 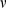 should scale (at the least) with 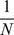. However, even if the cost terms scale with 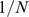, they will still dominate the network dynamics. For instance, the threshold, Eqn. (7) becomes independent of the output kernel, while the contribution of fast lateral connections becomes negligible. In practice, this causes the performance to degrade quickly with increasing network size. A better choice is to require 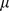 and 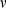 to scale with 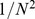, keeping the relative contribution of the kernel and cost to each neuron's dynamics fixed. With such scaling, large networks can still track the variable while the performance increase with network size.

Given the scaling of the output kernels and 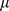, the threshold 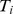 scales with 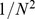, compare Eqn. (7). In turn, since the voltage is bounded by the threshold from above (and bounded from below due to the existence of neurons with opposing kernels; see also below), the voltage 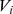 also scales with 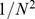. Accordingly, in a large network, the first, voltage-dependent term in Eqn. (36) scales with 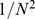, as do the terms 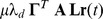 and 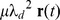. In contrast, the terms 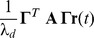 and 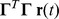 represent a sum over all neurons in the population, and thus scale with 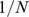, similar to the inputs 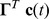. For large networks, we can therefore neglect the terms that scale with 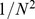. We note that none of the terms involving delta functions (i.e. 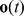) can be neglected. We keep a generic leak term, 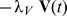, although the term is essentially irrelevant in large networks, and may be detrimental in very small ones (e.g., less than 10 neurons). Hence, we approximate Eqn. (36) by
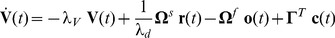
(37)with
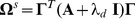
(38)

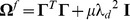
(39)Since 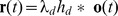 and since 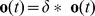, we can define the effective connectivities
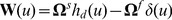
(40)to obtain the voltage equation
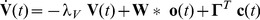
(41)which is the vectorized version of Eqn. (8) without the noise term.

### Precision of the network estimate and comparison with stochastic neural models

#### The predictive coding network

For simplicity, we will first discuss the homogeneous network, with one population of 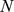 identical neurons, and no cost terms. A complete analytical solution of the homogeneous network is provided in Text S1. If the network is sufficiently large, we can replace the spike trains of the individual neurons by their population firing rate as shown in the Text S1. Here, we will furthermore assume that the variable 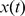 being tracked by the neurons is a constant. In this case, the network will produce a readout 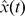 that is approximately constant as well, so that 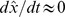. Solving the read-out equation for the rate, we therefore obtain
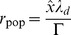
(42)where we neglected time since all variables are constants. Note use of the label “pop” which should remind us that this is the *population* firing rate, i.e., the sum of the firing rates of the individual units. These latter firing rates are simply given by dividing the population firing rate through the number of neurons 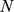 (since all units are equal) so that
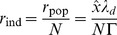
(43)To keep these firing rates constant as the size of the network increases, the kernels 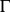 should therefore scale as 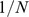.

To estimate the precision of the network readout, we note that the predictive coding scheme prescribed through the loss function implies that 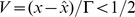, if the costs are negligible. Furthermore, since the neurons are firing at a constant rate, the voltage is bounded from below by the reset potential 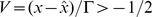. This bound holds in the limit of (infinitely) small noise. In turn, the readout is also bounded so that
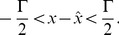
(44)The standard deviation of the network estimate, 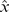, in the time window of the decoder 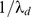 is thus strictly bounded by 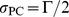, which, as stated previously, should scale with 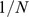 to maintain the same firing rate in the neurons. Accordingly, the standard deviation of the network estimate scales with
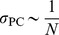
(45)as the network size increases.

#### The equivalent Poisson network

Let us consider now a set of 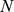 neurons firing spikes at the same rate 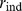 according to a homogeneous Poisson process. The minimal variance of an estimator based on measuring these neuron's responses in a time window 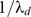 is given by the Cramer-Rao bound [47]

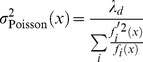
(46)where 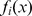 are the tuning curves of the neurons, i.e., their firing rate as a function of the encoded variable 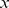. If we assume the best-case scenario, 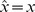, then the tuning curves are simply given by Eqn. (43), so that
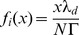
(47)Hence, the Cramer-Rao bound becomes
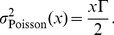
(48)As before, the kernels need to scale with 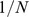, so that in this case the standard deviation scales with
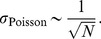
(49)


#### The general case

The exact analytical results cannot be readily extended to the representation of multi-dimensional variables, 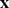, since the tuning curves of the individual neurons, 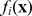, depend on the exact spatial configuration of the kernels. Here, we use instead arguments on how the kernels 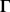 and the errors scale with the size of the network (assuming fixed firing rates and decoder leak). For simplicity of argumentation, we will assume that 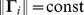 for all 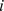, so that all output kernels have the same norm, and we can again neglect the cost term.

In the case of a multi-dimensional variable, the firing rule ensures that the projection of the prediction error, 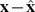, on any kernel 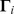 in the population does not exceed half of the kernel norm. Thus, if we place ourselves along the direction of any kernel, the projection of the prediction error is bounded by
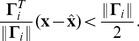
(50)As argued above, to keep the firing rates of the individual neurons constant, the kernels need to scale with 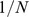 as the size of the network increases. Hence,
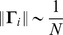
(51)and the projection of the error on each kernel direction thus scales with 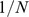. Let us assume that the kernels are sufficiently dense to cover all quadrant of the 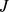-dimensional variable space. In this case, there is at least one kernel in each of the subspaces formed by all possible combinations of signs for the components 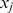. The minimum number of neurons is therefore 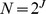. In each quadrant, there is then a kernel that will cause its associated neuron to fire as soon as the dot-product of this kernel with the error exceeds half the kernel norm. Consequently, the error is strictly bounded in a hypersphere centered on the origin whose radius is bounded by the constant kernel norm, Eqn. (50). Thus, as before, the standard deviation of the prediction error scales with the kernels and thus with
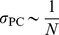
(52)


Let us now consider a population of 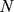 independent Poisson neurons representing the multi-dimensional variable 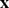. The minimal variance, 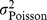, of the estimation errors are given by the inverse of the Fisher information matrix. The precise formula does not matter, though we note that this variance scales with 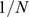. Thus, as before, we obtain
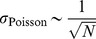
(53)and we can conclude that the ratio of the network estimation error to the estimation error incurred by 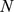 independent Poisson neurons with similar rates rates will always scale as 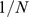.

### Rate model matching the firing rate of the homogeneous integrator network

In the homogeneous integrator network (with low noise and small costs), the membrane potentials of neurons with identical kernels are approximately equal, which allows us to write down an analytical solution (see Text S1). Briefly, the population inter-spike interval, i.e. the interval between two successive spikes from any neuron, corresponds to the time it takes for this “common” membrane potential to rise from the reset potential 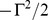 to the threshold 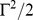. We call this time period an “integration cycle”. Note that this interval is typically much shorter than the ISI of an individual neuron or the time constant of the decoder. During this short time interval, the leak term 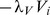 can be neglected, and the derivative of the membrane potential, 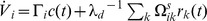, is approximately constant. The population ISI is thus simply given by the time 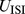 it takes to integrate from the reset, 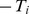, to the threshold, 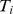, so that 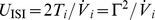. All neurons with the same kernel have identical firing rates, and, since only half of the population is spiking at any value of 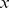 (in the limit of small noise), the firing rates of individual neurons are equal to the population firing divided by 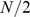. Thus, the firing rate of each neuron can be approximated as 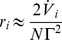.

To construct the Poisson generator network, we removed the fast connections (but not the slow connections) and replaced the LIF neurons by Poisson spike generators with the same instantaneous firing rate, i.e., 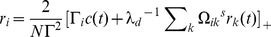. The resulting recurrent network roughly matches the instantaneous firing rates (but not the performance) in the LIF network. The match could be enhanced, for example by adding a small baseline firing rate or a refractory period; However, these changes can only decrease the performance of the Poisson rate model.

### Measuring GLM filters

To obtain the 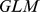 filters in the integrator network (Fig. 7 F), we performed the following procedure: The inhomogeneous integrator was driven by an input 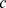 sampled from Gaussian white noise (with mean 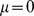, standard deviation 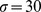) and convolved by an exponential filter of width 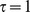
ms. The spike trains of the ten “recorded” neurons were modeled as independent Poisson processes with instantaneous firing rates
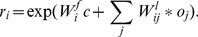
(54)The feed-forward weights 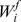 and lateral filters 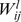 were estimated by maximizing the log-likelihood of the spike trains, following the method of [28]. Briefly, the filters were discretized in 500 time bins of 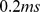, and conjugate gradient ascent of the log likelihood was performed on the value of the filters in each time bin for the equivalent of 5 hours of recording.

### Measuring the coefficient of variation

The 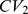 of a spike train is defined as
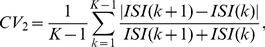
(55)where 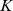 is the total number of spike in the spike train, and 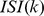 is the duration of the 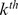 inter-spike interval. The 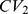 reported in the paper are the value of 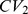 measured in each neuron and averaged over the population.

## Supporting Information

Text S1In the supporting Text S1, we address several issues regarding the biological plausibility of our approach and the generality of our results. In particular, we demonstrate that the networks can be separated into purely excitatory and inhibitory neurons. We discuss the problem of perturbing synaptic weights and show that the networks are robust to synaptic failures and to noise in the lateral connections. We furthermore derive analytical results for the dynamics of a network with identical kernels tracking a scalar dynamical variable, 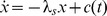, in the limit of high firing rates.(PDF)

## References

[1] Gold JI, Shadlen MN (2007) The neural basis of decision making. Annu Rev Neurosci 30: 535–574. 17600525 10.1146/annurev.neuro.29.051605.113038

[2] Major G, Tank D (2004) Persistent neural activity: prevalence and mechanisms. Curr Opin Neurobiol 14: 675–684. 15582368 10.1016/j.conb.2004.10.017

[3] Wolpert DM, Ghahramani Z (2000) Computational principles of movement neuroscience. Nat Neurosci 3: 1212–1217. 11127840 10.1038/81497

[4] Hertz J, Palmer R, Krogh A (1991) Introduction to the theory of neural computation Santa Fe Institute: Westview Press.

[5] Vogels TP, Rajan K, Abbott LF (2005) Neural network dynamics. Annu Rev Neurosci 28: 357–376. 16022600 10.1146/annurev.neuro.28.061604.135637

[6] Wang X-J (2008) Decision making in recurrent neuronal circuits. Neuron 60: 215–234. 18957215 10.1016/j.neuron.2008.09.034 PMC2710297

[7] Tolhurst D, Movshon J, Dean A (1982) The statistical reliability of signals in single neurons in cat and monkey visual cortex. Vision Res 23: 775–785. 10.1016/0042-6989(83)90200-6 6623937

[8] Shadlen MN, Newsome WT (1998) The variable discharge of cortical neurons: implications for connectivity, computation, and information coding. J Neurosci 18 (10) 3870–3896. 9570816 10.1523/JNEUROSCI.18-10-03870.1998 PMC6793166

[9] Compte A, Brunel N, Goldman-Rakic PS, Wang XJ (2000) Synaptic mechanisms and network dynamics underlying spatial working memory in a cortical network model. Cereb Cortex 10 (9) 910–923. 10982751 10.1093/cercor/10.9.910

[10] Machens CK, Romo R, Brody CD (2005) Flexible control of mutual inhibition: a neural model of two-interval discrimination. Science 307: 1121–1124. 15718474 10.1126/science.1104171

[11] Mainen ZF, Sejnowski TJ (1995) Reliability of spike timing in neocortical neurons. Science 268 (5216) 1503–1506. 7770778 10.1126/science.7770778

[12] Schneidman E, Freedman B, Segev I (1998) Ion channel stochasticity may be critical in determining the reliability and precision of spike timing. Neural Comput 10 (7) 1679–1703. 9744892 10.1162/089976698300017089

[13] Faisal AA, Selen LP, Wolpert DM (2008) Noise in the nervous system. Nat Rev Neurosci 9: 292–303. 18319728 10.1038/nrn2258 PMC2631351

[14] Softky WR, Koch C (1993) Noise in the nervous system. J Neurosci 13 (1) 334–350. 8423479 10.1523/JNEUROSCI.13-01-00334.1993 PMC6576320

[15] van Vreeswijk C, Sompolinsky H (1998) Chaotic balanced state in a model of cortical circuits. Neural Comput 10 (6) 1321–1371. 9698348 10.1162/089976698300017214

[16] Brunel N (2000) Dynamics of sparsely connected networks of excitatory and inhibitory neurons. J Comput Neurosci 8: 183–208. 10809012 10.1023/a:1008925309027

[17] Renart A, de la Rocha J, Bartho P, Hollender L, Parga N, et al (2010) The asynchronous state in cortical circuits. Science 327: 587–590. 20110507 10.1126/science.1179850 PMC2861483

[18] Tchumatchenko T, Malyshev A, Geisel T, Volgushev M, Wolf F (2010) Correlations and synchrony in threshold neuron models. Phys Rev Lett 104: 058102. 20366796 10.1103/PhysRevLett.104.058102

[19] Deneve S (2008) Bayesian spiking neurons i: inference. Neural Comput 20: 91–117. 18045002 10.1162/neco.2008.20.1.91

[20] Boerlin M, Deneve S (2011) Spike-based population coding and working memory. PLoS Comput Biol 7: e1001080. 21379319 10.1371/journal.pcbi.1001080 PMC3040643

[21] Leigh JR (2004) Control Theory: A Guided Tour. London, UK: Institution of Electrical Engineers.

[22] Wehr M, Zador AM (2003) Balanced inhibition underlies tuning and sharpens spike timing in auditory cortex. Nature 426: 442–6. 14647382 10.1038/nature02116

[23] Okun M, Lampl I (2008) Instantaneous correlation of excitation and inhibition during ongoing and sensory-evoked activities. Nat Neurosci 11: 535–537. 18376400 10.1038/nn.2105

[24] Haider B, Duque A, Hasenstaub AR, McCormick DA (2006) Neocortical network activity in vivo is generated through a dynamic balance of excitation and inhibition. J Neurosci 26: 4535–4545. 16641233 10.1523/JNEUROSCI.5297-05.2006 PMC6674060

[25] Shu Y, Hasenstaub A, McCormick DA (2003) Turning on and off recurrent balanced cortical activity. Nature 423: 288–293. 12748642 10.1038/nature01616

[26] Gentet LJ, Avermann M, Matyas F, Staiger JF, Petersen CCH (2010) Membrane potential dynamics of GABAergic neurons in the barrel cortex of behaving mice. Neuron 65: 422–435. 20159454 10.1016/j.neuron.2010.01.006

[27] Maass W, Natschlager T, Markram H (2002) Real-time computing without stable states: a new framework for neural computation based on perturbations. Neural Comput 14 (11) 2531–60. 12433288 10.1162/089976602760407955

[28] Pillow JW, Shlens J, Paninski L, Sher A, Litke AM, et al (2008) Spatiotemporal correlations and visual signalling in a complete neuronal population. Nature 454 (7207) 995–999. 18650810 10.1038/nature07140 PMC2684455

[29] Seung H (1996) How the brain keeps the eyes still. Proc Natl Acad Sci USA 93 (23) 13339–13344. 8917592 10.1073/pnas.93.23.13339 PMC24094

[30] Eliasmith C (2005) A unified approach to building and controlling spiking attractor networks. Neural Comput 17 (6) 1276–1314. 15901399 10.1162/0899766053630332

[31] Machens CK, Brody CD (2008) Design of continuous attractor networks with monotonic tuning using a symmetry principle. Neural Comput 20: 452–485. 18047414 10.1162/neco.2007.07-06-297

[32] Batista A, Buneo C, Snyder L, Andersen R (1999) Reach plans in eye-centered coordinates. Science 285: 257–60. 10398603 10.1126/science.285.5425.257

[33] Snyder L, Grieve K, Brotchie P, Andersen R (1998) Separate body- and world-referenced representations of visual space in parietal cortex. Nature 394: 887–91. 9732870 10.1038/29777

[34] Druckmann S, Chklovskii D (2010) Over-complete representations on recurrent neural networks can support persistent percepts. Advances in Neural Information Processing Systems 23: 541–549 (Lafferty J, Williams CKI, Shawe-Taylor J, Zemel R, Culotta A, editors).

[35] Ecker AS, Berens P, Keliris GA, Bethge M, Logothetis NK, Tolias AS (2007) Decorrelated neuronal firing in cortical microcircuits. Science 327: 584–587. 10.1126/science.1179867 20110506

[36] Roudi Y, Latham PE (2007) A balanced memory network. PLoS Comput Biol 3 (9) 1679–1700. 17845070 10.1371/journal.pcbi.0030141 PMC1971123

[37] Bourdoukan R, Barrett DGT, Machens CK, Deneve S (2012) Learning Optimal Spike-based Representations. Advances in Neural Information Processing Systems 25: 2294–2302.

[38] Abbott L, Dayan P (1999) The effect of correlated variability on the accuracy of a population code. Neural Computation 11: 91–101. 9950724 10.1162/089976699300016827

[39] Zohary E, Newsome WT (1994) Correlated neuronal discharge rate and its implication for psychophysical performance. Nature 370: 140–143. 8022482 10.1038/370140a0

[40] Koulakov A, Raghavachari S, Kepecs A, Lisman JE (2002) Model for a robust neural integrator. Nature Neurosci 5 (8) 775–782. 12134153 10.1038/nn893

[41] Goldman MS, Levine JH, Major G, Tank DW, Seung HS (2003) Robust persistent neural activity in a model integrator with multiple hysteretic dendrites per neuron. Cerebral cortex 13 (11) 1185–1195. 14576210 10.1093/cercor/bhg095

[42] Cain N, Barreiro AK, Shadlen M, Shea-Brown E (2013) Neural integrators for decision making: a favorable tradeo_ between robustness and sensitivity. J Neurophysiol 109 (10) 2542–59. 23446688 10.1152/jn.00976.2012 PMC3653050

[43] Moreau L, Sontag E (2003) Balancing at the border of instability. Physical Rev E 68 (2) 020901. 10.1103/PhysRevE.68.020901 14524945

[44] Renart A, Song P, Wang X-J (2003) Robust spatial working memory through homeostatic synaptic scaling in heterogeneous cortical networks. Neuron 38 (3) 473–485. 12741993 10.1016/s0896-6273(03)00255-1

[45] Goldman MS (2009) Memory without feedback in a neural network. Neuron 61 (4) 621–34. 19249281 10.1016/j.neuron.2008.12.012 PMC2674525

[46] Li S, Goldman MS (2013) Balanced cortical microcircuitry for maintaining information in working memory. Nat Neurosci 16: 1306–1314. 23955560 10.1038/nn.3492 PMC3772089

[47] Brunel N, Nadal J (1998) Mutual information, Fisher information, and population coding. Neural Comput 10 (7) 1731–57. 9744895 10.1162/089976698300017115

