# Supplementary material for: Predictive Coding of Dynamical Variables in Balanced Spiking Networks
Source: PLoS Comput Biol. 2013 Nov 14;9(11):e1003258. doi: 10.1371/journal.pcbi.1003258 (PMC3828152; doi:10.1371/journal.pcbi.1003258)
Supplement: Text S1 — In the supporting Text S1, we address several issues regarding the biological plausibility of our approach and the generality of our results. In particular, we demonstrate that the networks can be separated into purely excitatory and inhibitory neurons. We discuss the problem of perturbing synaptic weights and show that the networks are robust to synaptic failures and to noise in the lateral connections. We furthermore derive analytical results for the dynamics of a network with identical kernels tracking a scalar dynamical variable, , in the limit of high firing rates. (PDF) [file pcbi.1003258.s001.pdf]

Text S1

Martin Boerlin, Christian K. Machens, Sophie Denève

June 12, 2013

Brief Summary

In Sections 1, 2, and 3, we address several issues regarding the biological plausibility of our approach and the generality of our results. In particular, we demonstrate that the networks can be separated into purely excitatory and inhibitory neurons. We discuss the problem of perturbing synaptic weights and show that the networks are robust to synaptic failures and to noise in the lateral connections. In Section 4, we derive analytically the dynamics of a network with identical kernels tracking a scalar dynamical variable, $\dot{x} = -\lambda_s x + c(t)$, in the limit of high firing rates.

Contents

1 Towards biological plausibility: the necessity of linear and quadratic costs 3

1.1 The "ping-pong" effect and raising the threshold 3

1.2 The "smallest-kernel-wins" effect and decreasing the reset 3

2 Towards biological plausibility: Dale's law 5

2.1 Separating excitation and inhibition 5

2.2 Dale's law and the kernel signs 7

3 Robustness to perturbations: connection noise and synaptic failures 9

4 The homogeneous, leaky integrator network: Analytical results 14

4.1 Reduction to a single neuron 14

4.2 The integrate-and-fire neuron 17

4.3 Solution of the integrate-and-fire neuron 18

4.4 The limit of high firing rates 19

4.5 Robustness to perturbations: imbalances in excitation and inhibition 20

1 Towards biological plausibility: the necessity of linear and quadratic costs

Without any cost term, the network performs as advertised, whereby the read-outs, $\hat{\mathbf{x}}$, generate the desired dynamics, $\mathbf{x}$. However, the solutions found with the greedy optimization procedure can be biologically unrealistic. For instance, some neurons may fire at extremely high rates while others may stay completely silent. Here we discuss these potential problems, explain how they arise, and show how they can be solved through cost terms that penalize high firing rates.

1.1 The "ping-pong" effect and raising the threshold

Let us consider a network with output kernels $\mathbf{\Gamma}_i$ of equal length, yet pointing in opposite directions. Without any costs, a spike from one neuron (e.g. with kernel $\Gamma_i = 1$) will immediately excite any neuron with a kernel pointing in the opposite direction (e.g., with kernel $\Gamma_i = -1$). This excitation will bring these secondary neurons exactly to their firing threshold (in the limit of zero noise) or will make it highly likely that they fire (for finite noise). In turn, firing of these secondary neurons will bring the first neuron to its threshold, as well as all neurons with a similar kernel. Hence, all neurons with opposing kernels become engaged in rapidly firing volleys of spikes in fast succession, just as ping-pong players in a fast game. This problem is illustrated for two example neurons in Supplementary Fig. 1A. With only two neurons, the effect may eventually die out; however, if there are many neurons with similar output kernels of opposite signs, the effect tends to propagate and can eventually encompass the whole network. This outcome is an unfortunate by-product of the greedy optimization procedure, in which spikes are fired to compensate for current errors, but without considering their impact for future time points. Thankfully, including either a linear or quadratic cost in the loss function prevents the ping-pong dynamics since both of them elevate the firing threshold. Once the firing threshold is elevated, the spike of a neuron does not immediately trigger a spike in neurons with opposite kernels (see Supplementary Fig. 1A). [1]

1.2 The "smallest-kernel-wins" effect and decreasing the reset

A separate problem occurs whenever kernels have different sizes, i.e., when their norms differ from neuron to neuron. Consider two neurons whose output kernels point in the same direction but have different sizes. In the absence of noise, the neuron with the smaller kernel will always reach threshold first, fire, and thereby reset itself and the other neuron. Consequently, the neuron with the smaller kernel will "foot the whole bill" and fire at a high rate, while the neuron with the larger kernel will stay silent. This "smallest-kernel-wins" problem is illustrated in Supplementary Fig. 1B.[2] Note that the distribution of firing between the two neurons is a perfectly reasonable solution since choosing the smallest kernel minimizes the mean-squared error. Even if a linear cost term is introduced, this solution persists, since a linear cost term only penalizes the *global* firing rate, i.e. the total number of spikes fired by all neurons, without taking into account the division of labor amongst different neurons.

To distribute the firing more equally among neurons, we make use of the quadratic cost term, and set $\mu > 0$. The quadratic cost term penalizes the "smallest-kernel-wins" scenario since the sum of squared firing rates will be lower if all neurons participate in the representation of the information.

[1] The ping-pong effect can also be strongly decreased or even eliminated by the ad hoc addition of a refractory period.

[2] This problem does not occur in the main paper since all kernels were normalized in the simulations.

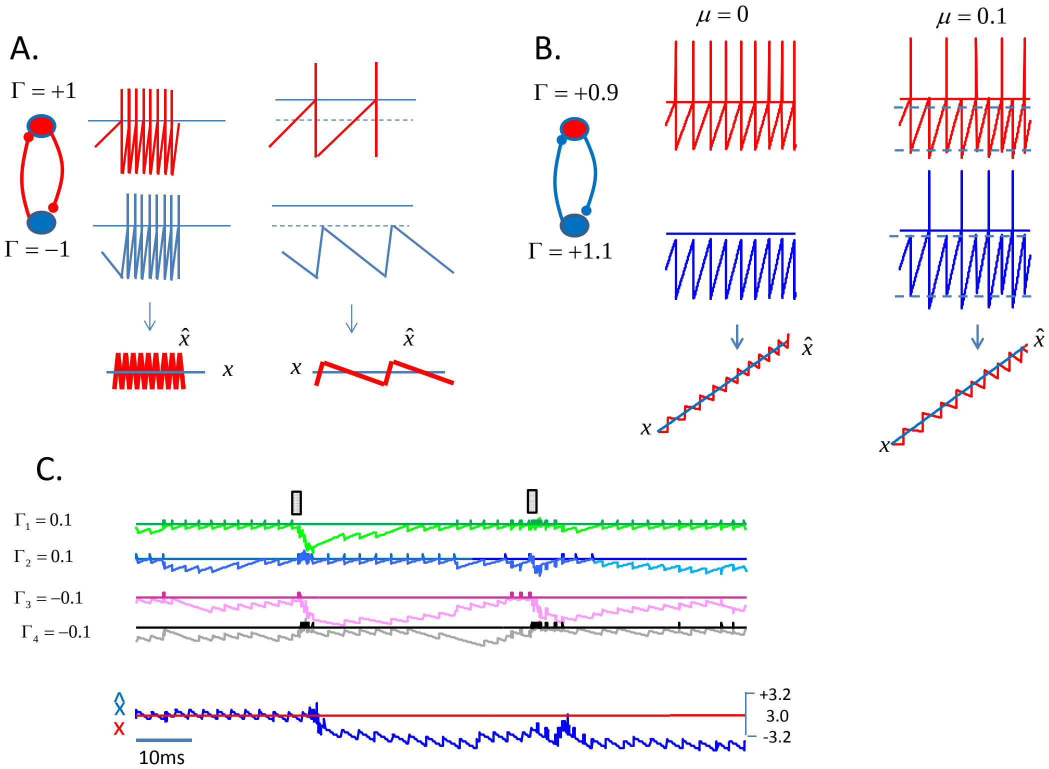

Supplementary Figure 1: Robustness of the network. **A.** Illustration of the "ping pong"-effect. If $\nu = 0$, a spike from a neuron with a positive kernel ($\Gamma_i = 1$) will bring a neuron with an opposite (negative) kernel ($\Gamma_i = -1$) exactly to its threshold, thereby causing it to spike. In turn, the spike from the neuron with negative kernel will excite the neuron with positive kernel, causing it to spike again. The process repeats ad infinitum, and the corresponding micro-circuit fires at the maximal rate (left panel). Addition of the cost factor $\nu > 0$ brings the threshold from its original position (dashed) to a higher level (plain line). As a result, a spike does not bring a neuron with opposite kernel to its threshold. If x is positive, only the neuron with positive kernel fires. The membrane potential of the neuron with negative kernels oscillates below threshold. **B.** Illustration of the "smallest-kernel-wins" effect. If $\mu = 0$, a neuron with a smaller kernel ($\Gamma_i = 0.9$) will always fire before a neuron with a larger kernel ($\Gamma_i = 1.1$)) since its threshold is lower. Both neurons are reset by the same amount, and the process repeats, causing the neuron with the smaller kernel to fire in every oscillation cycle while the neuron with the larger kernel never fires. This problem is alleviated by inclusion of the quadratic cost ($\mu = 0.1$) which leads to a larger reset of each neuron, thereby giving the other neuron a chance to fire. **C.** Illustration of synaptic failures. The cost terms may be insufficient in the presence of synaptic failures. In the four-neuron integrator network, synaptic failures cause synchronous firing of neurons with similar kernels (grey rectangles). This in turn recruits the two neurons with opposite kernels, starting a two-spikes ping-pong game. In the four top panels, green, blue, magenta and black lines represent the membrane potentials of the four neurons shown in a time window of 200ms. The horizontal lines are their firing threshold and the vertical lines above threshold are their spikes. In the bottom panels, the red and blue lines represent respectively the variable x and its estimate $\hat{x}$ in the same time window.

For sufficiently large μ, even neurons with larger kernels are recruited periodically, if less often than smaller kernels. This regularizing influence of a quadratic cost term is well-known from standard regression techniques such as ridge regression. Mechanistically, the parameter μ is added to both the threshold and the reset. The change in the reset means that a neurons' spike has a stronger (inhibitory) effect on itself than other neurons. In turn, this soft refractory period forces the neuron to wait longer before it can spike again, thereby giving other neurons (with larger kernels) a chance to spike.

Note that the quadratic cost term solves both the "ping-pong" problem and the "smallest-kernel-wins" problem. For the toy examples presented in the main text, where all kernels were normalized, using cost term μ instead of ν does not significantly change the network performance or any of the simulation results. However, we found that the quadratic cost term made the network more robust to synaptic failure or to noise in the network connectivity (see next section).

2 Towards biological plausibility: Dale's law

2.1 Separating excitation and inhibition

In the main text, model units can both excite and inhibit other units, which is biologically implausible and violates Dale's law. Here, we explain how to separate the total population into groups of purely excitatory and purely inhibitory units without affecting the network performance (apart from an approximate doubling of the total number of output spike).

Naively, we could simply double the number of neurons: for each excitatory neuron, we could introduce an inhibitory neuron which receives exactly the same connections and which fires exactly at the same time as the excitatory neuron. In turn, we could use either the excitatory or the inhibitory neurons depending on whether we need to excite or inhibit a postsynaptic neuron. However, such a scheme would suggest that there should be equal numbers of excitatory and inhibitory neurons in a system, whose firing should be tightly synchronized, which is rarely the case.

To move beyond this naive solution and construct more flexible and plausible networks, we will introduce separate error functions for the excitatory and inhibitory neural populations, letting the excitatory population track the actual signal, $\mathbf{x}(t)$, while the "local" inhibitory population tracks the estimate of the excitatory population. This scheme readily provides two closely matched estimates, $\hat{\mathbf{x}}_E(t)$ and $\hat{\mathbf{x}}_I(t)$, one represented by the population of excitatory neurons, the other by the population of inhibitory neurons. Thus, we can use the excitatory or the inhibitory spike trains interchangeably depending on whether we need to excite or inhibit a population of postsynaptic neurons. Importantly, excitatory and inhibitory neurons need not be matched in number, can receive different connections, and will fire asynchronously with respect to each other.

To keep the notation brief, we will limit the inclusion of cost terms, and use matrix notation. Let us consider N_I inhibitory neurons and N_E excitatory neurons with output kernels $\mathbf{\Gamma}_{I,i}$, $i = 1 \dots N_I$, and $\mathbf{\Gamma}_{E,i}$, $i = 1 \dots N_E$, respectively. We assume that these kernels are sufficiently similar so that $\mathbf{\Gamma}_{X,i}^T \mathbf{\Gamma}_{Y,j} \geq 0$ for all $X, Y \in \{I, E\}$ and for all i, j. As in the main paper, we will write $\mathbf{\Gamma}_E$ and $\mathbf{\Gamma}_I$ for the matrices whose rows are composed of the respective kernels such that $\mathbf{\Gamma}_E = (\mathbf{\Gamma}_{E,1}, \mathbf{\Gamma}_{E,2}, \dots, \mathbf{\Gamma}_{E,N_E})$ and equivalently for $\mathbf{\Gamma}_I$. The similarity of the kernels then implies that all the entries of the matrix $\mathbf{\Gamma}_X^T \mathbf{\Gamma}_Y$ are positive. As above, we define variables for the convolution of the spike trains with the exponential kernels $h_d(\cdot)$, using the short-hands $\bar{\mathbf{o}}_E(t) = h_d * \mathbf{o}_E(t)$ and $\bar{\mathbf{o}}_I(t) = h_d * \mathbf{o}_I(t)$. In turn, we define the estimate derived from the excitatory spike trains, $\mathbf{o}_E(t)$, as $\hat{\mathbf{x}}_E(t) = \mathbf{\Gamma}_E \bar{\mathbf{o}}_E(t)$ and that derived from the inhibitory spike trains, $\mathbf{o}_I(t)$, as $\hat{\mathbf{x}}_I(t) = \mathbf{\Gamma}_I \bar{\mathbf{o}}_I(t)$.

In the first step, we ensure that the estimate of the inhibitory population $\hat{\mathbf{x}}_I$ closely tracks the estimate of the excitatory population $\hat{\mathbf{x}}_E$. This is done by minimizing their cumulative mean-squared error:

$$E_I(t) = \int_0^t du \left(\sum_{j=1}^{J} \left(\hat{x}_{E,j}(u) - \hat{x}_{I,j}(u) \right)^2 \right) \tag{S1}$$

Just as described previously, we derive a rule for inhibitory spikes performing a greedy minimization of this error. We define the membrane potentials as $\mathbf{V}_I = \mathbf{\Gamma}_I^T(\hat{\mathbf{x}}_E - \hat{\mathbf{x}}_I)$ which results in a network of integrate-and-fire neurons with membrane potential dynamics

$$\dot{\mathbf{V}}_I = -\lambda_d \mathbf{V}_I + \mathbf{\Gamma}_I^T \mathbf{\Gamma}_E \mathbf{o}_E - \mathbf{\Gamma}_I^T \mathbf{\Gamma}_I \mathbf{o}_I. \tag{S2}$$

Moreover, a spike is fired in neuron i whenever $V_{I,i} > \|\mathbf{\Gamma}_{I,i}\|^2/2$. As a result, inhibitory spikes maintain the error between $\hat{\mathbf{x}}_E(t)$ and $\hat{\mathbf{x}}_I(t)$ below the amplitude of an inhibitory kernel. Note that the equation obeys Dale's law: the input of the excitatory neurons, $\mathbf{\Gamma}_I^T \mathbf{\Gamma}_E \mathbf{o}_E$, is always positive and depolarizes the voltage, whereas the input of the inhibitory neurons, $-\mathbf{\Gamma}_I^T \mathbf{\Gamma}_I \mathbf{o}_I$, is always negative and hyperpolarizes the voltage.

Meanwhile, the membrane potential of the excitatory neurons minimizes another mean-squared error, this time between the variable and the excitatory estimate, penalized by a cost term corresponding to the sum of excitatory spikes:

$$E_E(t) = \int_0^t du \left(\sum_{j=1}^{J} \left(x_j(u) - \hat{x}_{E,j}(u) \right)^2 + \nu \sum_{i=1}^{N} \bar{o}_{E,i}(u) \right) \tag{S3}$$

As derived previously, this error term can be minimized greedily by setting the neuron's membrane potentials to $\mathbf{V}_E = \mathbf{\Gamma}_E^T(\mathbf{x} - \hat{\mathbf{x}}_E)$ and the thresholds to $T_i = \left(\|\mathbf{\Gamma}_{E,i}\|^2 + \nu\right)/2$. The dynamics of the membrane potentials is then given by:

$$\dot{\mathbf{V}}_E = -\lambda_V \mathbf{V}_E + \mathbf{\Gamma}_E^T(\mathbf{c} + \mathbf{A}\mathbf{x}) - \mathbf{\Gamma}_E^T \dot{\hat{\mathbf{x}}}_E \tag{S4}$$

where the leak term, $-\lambda_V \mathbf{V}_E$, has been added for biological realism. Unfortunately, replacing $\dot{\hat{\mathbf{x}}}_E$ by its expression, $\dot{\hat{\mathbf{x}}}_E = -\lambda_d \hat{\mathbf{x}}_E + \mathbf{\Gamma}_E \mathbf{o}_E$, would directly lead to a network violating Dale's law because the fast lateral fast connections between excitatory neurons would then be negative. In order to obtain a network that respect Dale's law, we approximate the membrane potential dynamics by exchanging the excitatory and inhibitory estimates. Specifically, we replace $\dot{\hat{\mathbf{x}}}_E$ by its inhibitory approximation, $\dot{\hat{\mathbf{x}}}_I = -\lambda_d \hat{\mathbf{x}}_I + \mathbf{\Gamma}_I \mathbf{o}_I$. In turn, we arrive at the following expression for the dynamics:

$$\dot{\mathbf{V}}_E = -\lambda_V \mathbf{V}_E + \mathbf{\Gamma}_E^T(\mathbf{c} + \mathbf{A}\mathbf{x}) + \lambda_d \mathbf{\Gamma}_E^T \hat{\mathbf{x}}_I - \mathbf{\Gamma}_E^T \mathbf{\Gamma}_I \mathbf{o}_I \tag{S5}$$

We now are certain that the fast connections weights, $-\mathbf{\Gamma}_E^T \mathbf{\Gamma}_I$, are consistent with Dale's law, since they originate from inhibitory neurons. Next, we need to take care of the signs of the slow connections which will be determined by how we replace $\mathbf{x}$ and $\hat{\mathbf{x}}_I$.

Let us first first consider the case for which $\mathbf{\Gamma}_i^T(\mathbf{A} + \lambda_d \mathbf{I})\mathbf{\Gamma}_j \geq 0$ for all kernel pairs. This will generally happen whenever the dynamics of the variable is slower than the decoder's time constant ($\|\mathbf{A}\| \ll \lambda_d$), since then $\mathbf{\Gamma}^T(\mathbf{A} + \lambda_d \mathbf{I})\mathbf{\Gamma} \approx \lambda_d \mathbf{\Gamma}^T \mathbf{\Gamma}$. Both the sensory integrator in the main paper, with $\lambda_s \ll \lambda_d$, and the arm controller fall into this category. Using the self-consistency arguments

$\mathbf{x} \approx \hat{\mathbf{x}}_E = \mathbf{\Gamma}_E \bar{\mathbf{o}}_E$ and $\hat{\mathbf{x}}_I \approx \hat{\mathbf{x}}_E = \mathbf{\Gamma}_E \bar{\mathbf{o}}_E$, we can approximate the voltage dynamics of the excitatory neurons as follows:

$$\dot{\mathbf{V}}_E = -\lambda_V \mathbf{V}_E + \mathbf{\Gamma}_E^T \mathbf{c} + \mathbf{\Gamma}_E^T (\mathbf{A} + \lambda_d \mathbf{I}) \mathbf{\Gamma}_E \bar{\mathbf{o}}_E - \mathbf{\Gamma}_E^T \mathbf{\Gamma}_I \mathbf{o}_I. \tag{S6}$$

Thus, in this case, "slow connections" are provided by the excitatory neurons while "fast connections" are provided by the inhibitory neurons Supplementary Fig. 2A. Note that the equations now satisfy Dale's law.

Next, we consider the case where the dynamics is faster than the decoder, i.e., $\mathbf{\Gamma}_i^T (\mathbf{A} + \lambda_d \mathbf{I}) \mathbf{\Gamma}_j \leq 0$. This will be the case if, e.g., eig(A) $\leq -\lambda_d$. The leaky sensory integrator with $\lambda_d < \lambda_s$ falls into this category. In this condition, the previous substitution of $\mathbf{x}$ by $\hat{\mathbf{x}}_E$ and $\hat{\mathbf{x}}_I$ by $\hat{\mathbf{x}}_E$ would lead to the excitatory neurons sending negative slow connections, since $\mathbf{\Gamma}_E^T (\mathbf{A} + \lambda_d \mathbf{I}) \mathbf{\Gamma}_E$ is now negative. Instead, we replace $\mathbf{x}$ by $\hat{\mathbf{x}}_I$ in Eq. (S5) and obtain the dynamics

$$\dot{\mathbf{V}}_E = -\lambda_V \mathbf{V}_E + \mathbf{\Gamma}_E^T \mathbf{c} + \mathbf{\Gamma}_E^T (\mathbf{A} + \lambda_d \mathbf{I}) \mathbf{\Gamma}_I \bar{\mathbf{o}}_I - \mathbf{\Gamma}_E^T \mathbf{\Gamma}_I \mathbf{o}_I \tag{S7}$$

Thus, in this case, both fast and slow connections are provided by the inhibitory neurons (Supplementary Fig. 2B). The equations again fulfill Dale's law.

These two networks illustrate how one can use the ideas laid out in the main text to construct quite flexible networks of inhibitory and excitatory neurons. The great advantage of these architectures is that they provide two closely matched estimates $\hat{\mathbf{x}}_E(t)$ and $\hat{\mathbf{x}}_I(t)$, represented by populations of excitatory and inhibitory neurons. Thus, we can use the excitatory or the inhibitory spike trains interchangeably, depending on whether the corresponding connections need to be positive or negative.[3]. We do not necessarily have to assume that all kernels are similar, as stated at the beginning of this section. To illustrate this in one particular example, we will now construct a network with populations of neurons featuring opposing kernels. The network described in the next section can be used to implement the homogeneous and inhomogeneous integrator networks presented in the main text.

2.2 Dale's law and the kernel signs

Let us consider another set of neurons with output kernels $\mathbf{\Phi}_X$, also correlated (i.e. $\mathbf{\Phi}_{X,i}^T \mathbf{\Phi}_{Y,j} \geq 0$ with $X, Y \in \{I, E\}$ and for any i, j) but pointing in a globally opposite direction (i.e. $\mathbf{\Phi}_{X,i}^T \mathbf{\Gamma}_{Y,j} \leq 0$ for all X, Y and i, j). We will use superscripts Γ and Φ to distinguish the two subpopulations and define the excitatory and inhibitory estimates of the opposite population as $\hat{\mathbf{x}}_E^\Phi(t) = \mathbf{\Phi}_E \bar{\mathbf{o}}_E^\Phi(t)$ and $\hat{\mathbf{x}}_I^\Phi(t) = \mathbf{\Phi}_I \bar{\mathbf{o}}_I^\Phi(t)$. The membrane potential of the inhibitory neurons are minimizing the "local" prediction errors between excitatory and inhibitory estimates in the two sub-populations, i.e.,

$$E_I^\Gamma(t) = \int_0^t du \left(\sum_{j=1}^J \left(\hat{x}_{E,j}^\Gamma(u) - \hat{x}_{I,j}^\Gamma(u) \right)^2 \right) \tag{S8}$$

$$E_I^\Phi(t) = \int_0^t du \left(\sum_{j=1}^J \left(\hat{x}_{E,j}^\Phi(u) - \hat{x}_{I,j}^\Phi(u) \right)^2 \right). \tag{S9}$$

[3] Although more involved, we could therefore also work with matrices $\mathbf{A}$ for which slow connections $\mathbf{\Gamma}_i^T (\mathbf{A} + \lambda_d \mathbf{I}) \mathbf{\Gamma}_j$ can be either positive or negative depending on the neural pair (i, j)

Using greedy minimization of these two functions, we obtain the voltages $\mathbf{V}_I^\Gamma = \boldsymbol{\Gamma}_I^T(\hat{\mathbf{x}}_E^\Gamma - \hat{\mathbf{x}}_I^\Gamma)$ and $\mathbf{V}_I^\Phi = \boldsymbol{\Phi}_I^T(\hat{\mathbf{x}}_E^\Phi - \hat{\mathbf{x}}_I^\Phi)$ and the threshold rules $V_{I,i}^\Gamma > \|\boldsymbol{\Gamma}_{I,i}\|^2/2$ and $V_{I,i}^\Phi > \|\boldsymbol{\Phi}_{I,i}\|^2/2$. The resulting dynamics are (compare Eq. (S2))

$$\dot{\mathbf{V}}_I^\Gamma = -\lambda_d \mathbf{V}_I^\Gamma + \boldsymbol{\Gamma}_I^T \boldsymbol{\Gamma}_E \mathbf{o}_E^\Gamma - \boldsymbol{\Gamma}_I^T \boldsymbol{\Gamma}_I \mathbf{o}_I^\Gamma \tag{S10}$$

$$\dot{\mathbf{V}}_I^\Phi = -\lambda_d \mathbf{V}_I^\Phi + \boldsymbol{\Phi}_I^T \boldsymbol{\Phi}_E \mathbf{o}_E^\Phi - \boldsymbol{\Phi}_I^T \boldsymbol{\Phi}_I \mathbf{o}_I^\Phi \tag{S11}$$

In contrast to the inhibitory neurons which track the output of each population of kernels separately (hence providing "local inhibition"), excitatory neurons minimize the distance between the variable $\mathbf{x}$ and the "global" network estimate provided by combining both kernel populations. Thus, the membrane potentials of the excitatory neurons minimize the mean-squared error between the variable $\mathbf{x}$ and a global network estimate corresponding to $(\hat{\mathbf{x}}_E^\Gamma + \hat{\mathbf{x}}_E^\Phi)$,[4]

$$E_E(t) = \int_0^t du \left(\sum_{j=1}^{J} \left(x_j(u) - \hat{x}_{E,j}^\Gamma(u) - \hat{x}_{E,j}^\Phi(u) \right)^2 + \nu \sum_{i=1}^{N/2} \bar{o}_{E,i}^\Gamma(u) + \nu \sum_{i=N/2+1}^{N} \bar{o}_{E,i}^\Phi(u) \right). \tag{S12}$$

Using the derivation described above, the excitatory membrane potentials of the "Γ" population ($\mathbf{V}_E^\Gamma$) and the "Φ" population ($\mathbf{V}_E^\Phi$) are given by $\mathbf{V}_E^\Gamma = \boldsymbol{\Gamma}_E^T(\mathbf{x} - \hat{\mathbf{x}}_E^\Gamma - \hat{\mathbf{x}}_E^\Phi)$ and $\mathbf{V}_E^\Phi = \boldsymbol{\Phi}_E^T(\mathbf{x} - \hat{\mathbf{x}}_E^\Gamma - \hat{\mathbf{x}}_E^\Phi)$, respectively. Their dynamics is described by the equations

$$\dot{\mathbf{V}}_E^\Gamma = -\lambda_V \mathbf{V}_E^\Gamma + \boldsymbol{\Gamma}_E^T(\mathbf{c} + \mathbf{A}\mathbf{x}) - \boldsymbol{\Gamma}_E^T(\dot{\hat{\mathbf{x}}}_E^\Gamma + \dot{\hat{\mathbf{x}}}_E^\Phi) \tag{S13}$$

$$\dot{\mathbf{V}}_E^\Phi = -\lambda_V \mathbf{V}_E^\Phi + \boldsymbol{\Phi}_E^T(\mathbf{c} + \mathbf{A}\mathbf{x}) - \boldsymbol{\Phi}_E^T(\dot{\hat{\mathbf{x}}}_E^\Gamma + \dot{\hat{\mathbf{x}}}_E^\Phi) \tag{S14}$$

Next, we approximate $\dot{\hat{\mathbf{x}}}_E^\Gamma$ by the estimate of the local inhibitory population, $\dot{\hat{\mathbf{x}}}_I^\Gamma$, in the equations for the "Γ"-population. We also replace $\dot{\hat{\mathbf{x}}}_E^\Phi$ by $\dot{\hat{\mathbf{x}}}_I^\Phi$ in the expression for the "Φ"-population. This gives us:

$$\dot{\mathbf{V}}_E^\Gamma = -\lambda_V \mathbf{V}_E^\Gamma + \boldsymbol{\Gamma}_E^T(\mathbf{c} + \mathbf{A}\mathbf{x}) + \lambda_d \boldsymbol{\Gamma}_E^T(\hat{\mathbf{x}}_I^\Gamma + \hat{\mathbf{x}}_E^\Phi) - \boldsymbol{\Gamma}_E^T \boldsymbol{\Gamma}_I \mathbf{o}_I^\Gamma - \boldsymbol{\Gamma}_E^T \boldsymbol{\Phi}_E \mathbf{o}_E^\Phi \tag{S15}$$

$$\dot{\mathbf{V}}_E^\Phi = -\lambda_V \mathbf{V}_E^\Phi + \boldsymbol{\Phi}_E^T(\mathbf{c} + \mathbf{A}\mathbf{x}) + \lambda_d \boldsymbol{\Phi}_E^T(\hat{\mathbf{x}}_I^\Phi + \hat{\mathbf{x}}_E^\Gamma) - \boldsymbol{\Phi}_E^T \boldsymbol{\Phi}_I \mathbf{o}_I^\Phi - \boldsymbol{\Phi}_E^T \boldsymbol{\Gamma}_E \mathbf{o}_E^\Gamma \tag{S16}$$

At this stage, the fast connections have the right signs depending on whether they originate from excitatory neurons (all components of $-\boldsymbol{\Gamma}_E^T \boldsymbol{\Phi}_E$ and $-\boldsymbol{\Phi}_E^T \boldsymbol{\Gamma}_E$ are indeed positive) or from inhibitory neurons (all components of $-\boldsymbol{\Gamma}_E^T \boldsymbol{\Gamma}_I$ and $-\boldsymbol{\Phi}_E^T \boldsymbol{\Phi}_I$ are indeed negative).

We now need to take care of the slow connections. As before, the exact structure of slow lateral connections will depend on whether the dynamics of the variable is faster or slower than the decoder. If $\|\mathbf{A}\| \ll \lambda_d$, slow connections among neurons with similar selectivity, $\boldsymbol{\Gamma}^T(\mathbf{A}+\lambda_d\mathbf{I})\boldsymbol{\Gamma}$ and $\boldsymbol{\Phi}^T(\mathbf{A}+\lambda_d\mathbf{I})\boldsymbol{\Phi}$, are excitatory, while slow connections between neurons of opposite selectivity, $\boldsymbol{\Gamma}^T(\mathbf{A}+\lambda_d\mathbf{I})\boldsymbol{\Phi}$ and $\boldsymbol{\Phi}^T(\mathbf{A}+\lambda_d\mathbf{I})\boldsymbol{\Gamma}$, are inhibitory. We thus combine the excitatory estimate from neurons with similar kernels and the inhibitory estimates from neurons with opposite kernels to implement the slow currents (Supplementary Fig. 2C). More precisely, we replace $\mathbf{x}$ and $\hat{\mathbf{x}}_I^\Gamma + \hat{\mathbf{x}}_E^\Phi$ by $\hat{\mathbf{x}}_E^\Gamma + \hat{\mathbf{x}}_I^\Phi$ in the Γ-population. Vice versa, we replace $\mathbf{x}$ and $\hat{\mathbf{x}}_I^\Phi + \hat{\mathbf{x}}_E^\Gamma$ by $\hat{\mathbf{x}}_E^\Phi + \hat{\mathbf{x}}_I^\Gamma$ in the Φ-population. The resulting network obeys Dale's law (Supplementary Fig. 2C).

$$\dot{\mathbf{V}}_E^\Gamma = -\lambda_V \mathbf{V}_E^\Gamma + \boldsymbol{\Gamma}_E^T\mathbf{c} + \boldsymbol{\Gamma}_E^T(\mathbf{A} + \lambda_d I)\boldsymbol{\Gamma}_E \bar{\mathbf{o}}_E^\Gamma + \boldsymbol{\Gamma}_E^T(\mathbf{A} + \lambda_d I)\boldsymbol{\Phi}_I \bar{\mathbf{o}}_I^\Phi - \boldsymbol{\Gamma}_E^T \boldsymbol{\Gamma}_I \mathbf{o}_I^\Gamma - \boldsymbol{\Gamma}_E^T \boldsymbol{\Phi}_E \mathbf{o}_E^\Phi \tag{S17}$$

$$\dot{\mathbf{V}}_E^\Phi = -\lambda_V \mathbf{V}_E^\Phi + \boldsymbol{\Phi}_E^T\mathbf{c} + \boldsymbol{\Phi}_E^T(\mathbf{A} + \lambda_d I)\boldsymbol{\Phi}_E \bar{\mathbf{o}}_E^\Phi + \boldsymbol{\Phi}_E^T(\mathbf{A} + \lambda_d I)\boldsymbol{\Gamma}_I \bar{\mathbf{o}}_I^\Gamma - \boldsymbol{\Phi}_E^T \boldsymbol{\Phi}_I \mathbf{o}_I^\Phi - \boldsymbol{\Phi}_E^T \boldsymbol{\Gamma}_E \mathbf{o}_E^\Gamma \tag{S18}$$

[4]Note that the individual estimates, $\hat{\mathbf{x}}_E^\Gamma$ or $\hat{\mathbf{x}}_E^\Phi$, no longer represent the variable $\mathbf{x}$—only their sum does!

If the dynamics is faster than the decoder, i.e. $\|\mathbf{A}\| \gg \lambda_d$, slow connections among neurons with similar selectivity $\mathbf{\Gamma}^T(\mathbf{A}+\lambda_d\mathbf{I})\mathbf{\Gamma}$ and $\mathbf{\Phi}^T(\mathbf{A}+\lambda_d\mathbf{I})\mathbf{\Phi}$ are inhibitory, while slow connections between neurons of opposite selectivity $\mathbf{\Gamma}^T(\mathbf{A}+\lambda_d\mathbf{I})\mathbf{\Phi}$ and $\mathbf{\Phi}^T(\mathbf{A}+\lambda_d\mathbf{I})\mathbf{\Gamma}$ are excitatory, we thus replace $\mathbf{x}$ by $\hat{\mathbf{x}}_I^\Gamma + \hat{\mathbf{x}}_E^\Phi$ in the Γ-population, and $\mathbf{x}$ by $\hat{\mathbf{x}}_I^\Phi + \hat{\mathbf{x}}_E^\Gamma$ in the Φ-population. The resulting network also obey's Dale's law (Supplementary Fig. 2D):

$$\dot{\mathbf{V}}_E^\Gamma = -\lambda_V \mathbf{V}_E^\Gamma + \mathbf{\Gamma}_E^T\mathbf{c} + \mathbf{\Gamma}_E^T(\mathbf{A}+\lambda_d I)\mathbf{\Gamma}_I\bar{\mathbf{o}}_I^\Gamma + \mathbf{\Gamma}_E^T(\mathbf{A}+\lambda_d I)\mathbf{\Phi}_E\bar{\mathbf{o}}_E^\Phi - \mathbf{\Gamma}_E^T\mathbf{\Gamma}_I\mathbf{o}_I^\Gamma - \mathbf{\Gamma}_E^T\mathbf{\Phi}_E\mathbf{o}_E^\Phi \quad \text{(S19)}$$

$$\dot{\mathbf{V}}_E^\Phi = -\lambda_V \mathbf{V}_E^\Phi + \mathbf{\Phi}_E^T\mathbf{c} + \mathbf{\Phi}_E^T(\mathbf{A}+\lambda_d I)\mathbf{\Phi}_I\bar{\mathbf{o}}_I^\Phi + \mathbf{\Phi}_E^T(\mathbf{A}+\lambda_d I)\mathbf{\Gamma}_E\bar{\mathbf{o}}_E^\Gamma - \mathbf{\Phi}_E^T\mathbf{\Phi}_I\mathbf{o}_I^\Phi - \mathbf{\Phi}_E^T\mathbf{\Gamma}_E\mathbf{o}_E^\Gamma \quad \text{(S20)}$$

Simulation results of an inhomogeneous perfect integrator and very leaky integrator networks are shown in Supplementary Fig. 3A,B. Here we took the same sets of kernels as the inhomogeneous network described in the main text. We separated each group of 200 collinear kernels into 150 excitatory and 50 inhibitory units. The total number of excitatory spikes is approximately the same as for the unseparated network, with approximately the same number of inhibitory spikes. The network performance is identical, tuning curves and ISI distributions of excitatory and inhibitory neurons are scaled versions of those reported in the main text.

This example demonstrates that we can use a population of "local" inhibitory neurons to track the output of sets of similar kernels, i.e., kernels with positive dot products. As a consequence, excitatory and inhibitory neurons do not need to be matched in number, fire asynchronously, or receive the same connections. Note, however, that the inhibitory population as a whole is always highly correlated with the excitatory population. An excitatory spike is systematically followed by an inhibitory spike (Supplementary Fig. 3C). This does not result in strong pair-wise correlations between spike trains, however, since a different pair of excitatory and inhibitory neurons fires in each integration cycle. Moreover, the apparent independence of excitatory and inhibitory spike trains masks an extreme correlation in membrane potential. Pairs of excitatory neurons receive synchronous excitatory inputs (from feedforward input c), synchronous inhibition (from the inhibitory population), and an EPSC (e.g., caused by a positive input $\mathbf{c}(t)$) is followed by an IPSC of matching amplitude (Supplementary Fig. 3D). Note that the separation of excitation and inhibition comes at the cost of a doubling in the number of output spikes (one inhibitory spike per excitatory spike, but not necessarily one inhibitory neuron for each excitatory neuron) for the same achieved precision.

3 Robustness to perturbations: connection noise and synaptic failures

Thanks to its error-correcting properties, the network is naturally insensitive to noise in the spike generation process, partial inactivation of neurons, or background synaptic noise. Indeed, predictive coding endows the network with the capacity to correct its own mistakes online (even if this is at the cost of additional spikes). However, other forms of neural noise, such as synaptic failures or mistuning of the synaptic connectivity can potentially be far more destructive since they disrupt the communication of the prediction errors between different neurons. At this point, we cannot provide a complete, analytical understanding of how such failures influence the network dynamics. However, in the following, we provide some "rules of thumb" and show how networks can cope with these types of noise.

To gain some initial insights into what happens during synaptic failures, consider two neurons with identical positive kernels $\Gamma_1 = \Gamma_2 = 0.1$ (green and blue cells in Supplementary Fig. 1C; we

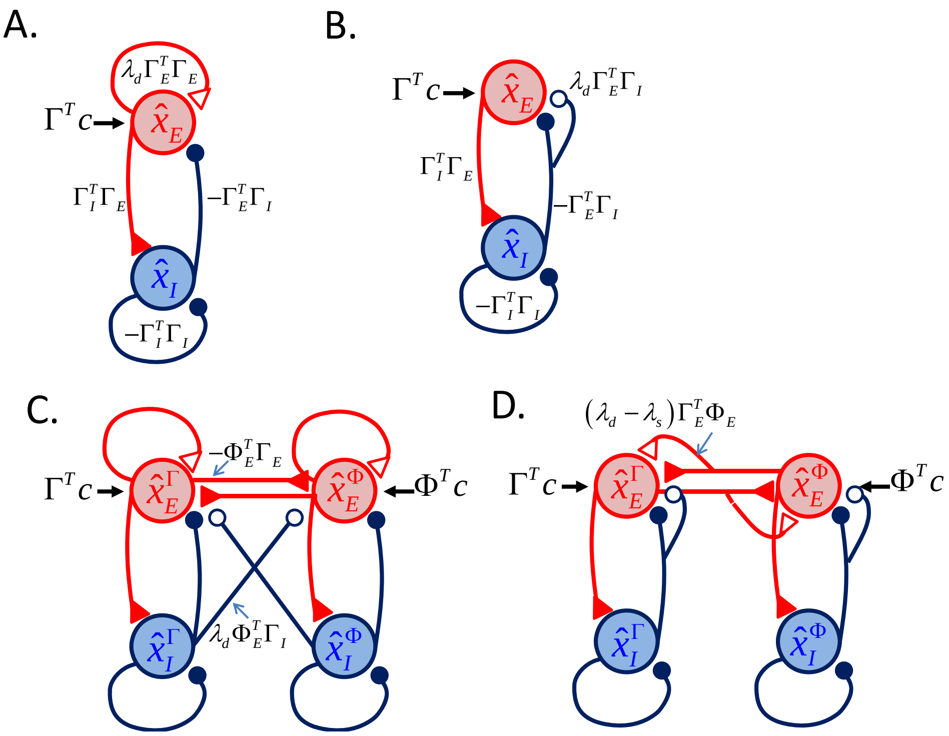

Supplementary Figure 2: Networks separating excitation from inhibition: structure. **A.** Integrator network ($A = \lambda_s$) and $|\lambda_s| \ll \lambda_d$ with collinear kernels. **B.** Very leaky integrator network, $|\lambda_s| > \lambda_d$. **C.** Integrator network with collinear and anti-collinear kernels. **D.** Very leaky network with collinear and anti-collinear kernels. Red triangles=excitatory connections. Blue circles=inhibitory connections. Filled connection endpoints: fast connections (instantaneous update of membrane potential). Unfilled connection endpoints: slow connections (currents with time constant λ_d).

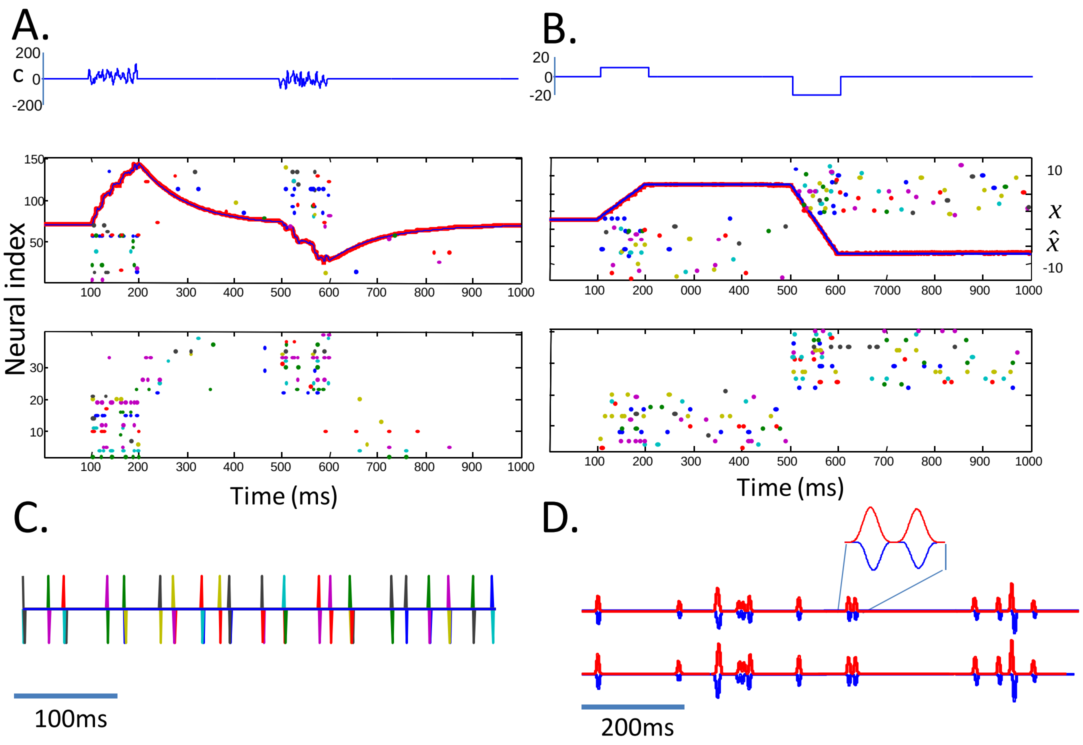

Supplementary Figure 3: Networks separating excitation from inhibition: simulation results. **A.** Very leaky integrator network (leaky integrator with $\lambda_s = 100$). Top panel: The command $c(t)$ consists of two pulses (one positive, one negative) corrupted by gaussian white noise. Middle panel: Variable (blue) and network estimate (red) overlaid on top of spike rasters from 140 excitatory neurons (bottom 70 lines: positive kernels, top 70 lines: negative kernels). Same legend as in Fig. 1C,D in the main text. Bottom panel: Spikes from 80 inhibitory neurons. Bottom 40: positive kernels, top 40: negative kernels. **B.** Integrator network (perfect integrator with $\lambda_s = 0$). Panels as in A. **C.** Excitatory spikes (top) and inhibitory spikes (bottom) emitted by the entire population during 500ms. Different colors represent spikes from different neurons, with possible repeats. Note that an inhibitory spike systematically follows an excitatory spike. **D.** Balance of excitation and inhibition. Here, we tested a network that purely tracks its input, i.e. $\lambda_s > \lambda_d$. We took the network in the rest state (no input) and gave pulses of excitatory input $c(t)$ at random times. The duration of the pulse was 1ms and the amplitude of the pulse was chosen uniformly between 500 and 1000. These inputs could be interpreted as EPSCs from presynaptic neurons. We show the excitatory and inhibitory currents received by 2 cells with collinear kernels. Currents were convolved by a gaussian window of STD $4ms$. Each IPSC is in fact composed of several inhibitory spikes (1 to 5) conveyed by fast inhibitory connections, since the pulses are strong enough to bring some neurons to their firing threshold. There are more inhibitory spikes caused by stronger input pulses, hence the correlation between EPSCs and IPSCs. Inset shows that IPSCs are slightly delayed compare to EPSCs (due to synaptic delays in the excitatory and inhibitory lateral connections).

ignore the other cells for the moment). These two neurons receive the same input and reach their firing threshold at approximately the same time. When one neuron reaches threshold first, it fires and normally inhibits the other neuron, such that only one spike is fired in each integration cycle. However, if the fast inhibitory synapse between these two neurons fails, then they will both fire, synchronously, within the same integration cycle (e.g. marked with grey rectangles in Supplementary Fig. 1C). The neurons then contribute two spikes (instead of one) to the decoder output $\hat{x}$ (blue line at the bottom of Supplementary Fig. 1C) so that the variable x is over-estimated (red line) by 0.1. The failure has thereby introduced a small error in the network estimate. Whether this error is forgotten or remembered will depend on the dynamics of the variable x. More precisely, if the synaptic failure encompasses both the fast and slow synapse of a neuron, then the error will influence the value of x on the right-hand-side of the dynamical equation, Eq. (1) in the article. In other words, a synaptic failure is then akin to adding a small noise term, scaled with the parameters $\mathbf{A}$, in the dynamical equation itself.

A positive estimation error may become problematic in another sense, however, if there are neurons with negative kernels in the network. For example, in Supplementary Fig. 1C, there are two inhibitory neurons with kernels $\Gamma_3 = \Gamma_4 = -0.1$ (grey and magenta lines). These neurons are excited by neuron 1 and 2. Thus, receiving synchronous spikes from neuron 1 and 2 will make them reach threshold and fire (i.e., they will detect the increase in prediction error and attempt to correct it), compensating for extra positive kernels with negative kernels. Unfortunately, this does not stop there. The two synchronous spikes from neuron 3 and 4 might in turn make both neuron 1 and 2 fire, which in turn might make both neuron 3 and 4 fire, starting a new ping-pong game with two "balls" instead of one (grey rectangles in Supplementary Fig. 1C). The variable can still be tracked quite precisely (compare red and blue lines), but with unnecessarily high firing rates. In this case, the synaptic failure has caused the reoccurrence of the "ping-pong" effect.

One way to avoid this problem is to increase the cost terms ν and/or μ until the resulting increases in firing threshold prevent synchronous spikes from recruiting neurons with opposite kernels. Unfortunately, increasing the cost also decreases the precision of the network, as expected from the error function. This tradeoff is illustrated in Supplementary Fig. 4A–C. Thus, the homogenous integrator with 10% synaptic failure can track the variable precisely, but at the cost of very high firing rates (Supplementary Fig. 4A,B). To avoid the ping-pong effect and decrease firing rates, we increase the cost terms to $\nu = 0.03$, $\mu = 0.03$ in Supplementary Fig. 4C. Unfortunately, at such high costs the network under-estimates the variable and brings the integrator time constant from almost infinite to about four seconds.

However, note that the "ping-pong" effect occurs only because some kernels point in exactly opposite directions. This is a consequence of the symmetrical architecture we used for the neural integrator. We could simply construct networks that perform the same computation but have no kernels pointing in opposite directions. These networks are more robust to synaptic failure. For example, this can be achieved by assuming that in addition to tracking the variable $\mathbf{x}$, the network enforces a constant population firing rate. In other words, in addition to $x_{1\ldots J}$, the population tracks a dummy variable x_{J+1} such that $\dot{x}_{J+1} = -\lambda_d x_{J+1} + \sqrt{\rho}\lambda_d r_o$, where r_o is the population firing rate and ρ is a small positive constant. Each neuron contributes to estimating this dummy variable with identical weight, $\Gamma_{i,J+1} = \sqrt{\rho}$, such that $\dot{\hat{x}}_{J+1} = -\lambda_d \hat{x}_{J+1} + \sqrt{\rho}\sum_i o_i$, and thus $\hat{x}_{J+1} = \sqrt{\rho}\sum_i \bar{o}_i$. The new error function corresponds to

$$E(t) = \int_0^t du \left(\sum_{j=1}^{J} \big(x_j(u) - \hat{x}_j(u)\big)^2 + \rho \Big(\sum_{i=1}^{N} \bar{o}_i(u) - r_o \Big)^2 \right). \tag{S21}$$

Here we omitted any additional linear and quadratic cost terms for the sake of simplicity. We also neglected initial conditions and replaced the dummy variable by its stable state $x_{J+1} = \sqrt{\rho} r_o$. Thus, in addition to minimizing the mean-squared error $\|\mathbf{x}_j(u) - \hat{\mathbf{x}}_j(u)\|^2$, the network maintains the population firing rate around r_o. With the addition of the dummy variable x_{J+1}, the new neural kernels are $\mathbf{\Gamma}_i = [\Gamma_{i1}, \Gamma_{i2}, \Gamma_{i3} \ldots, \Gamma_{iN}, \sqrt{\rho}]$. The new parameters of the dynamics are given by

$$\mathbf{A}' = \begin{pmatrix} A_{11} & A_{12} & \ldots & A_{1J} & 0 \\ A_{21} & A_{22} & \ldots & A_{2J} & 0 \\ \vdots & \vdots & & \vdots & \vdots \\ A_{J1} & A_{J2} & \ldots & A_{JJ} & 0 \\ 0 & 0 & \ldots & 0 & -\lambda_d \end{pmatrix} \qquad \mathbf{c}' = \begin{pmatrix} c_1 \\ c_2 \\ \vdots \\ c_J \\ \sqrt{\rho} r_o \end{pmatrix} \tag{S22}$$

The fast connections are given by $\Omega^f_{kl} = \sum_{j=1}^J \Gamma_{lj}\Gamma_{kj} + \rho$, and the threshold by $T_k = \sum_{j=1}^J \Gamma^2_{kj}/2 + \rho/2$. The slow connections are unchanged, since the dynamics of x_{J+1} is the same as the dynamics of the decoder. Finally, the input received by each neuron is given by $\sum_{j=1}^J \Gamma_{kj} c_j + \sqrt{\rho} r_o$. Thus, enforcing a small baseline firing rate is achieved by adding a positive constant ρ to all the fast connections in the network, a positive constant $\rho/2$ to all thresholds, and by providing a constant depolarizing current ρr_o to all neurons.

Note that due to the addition of the dummy weight $\Gamma_{i,J+1} = \rho$, the kernels do not point in opposite directions in $(J+1)$-D space. This, in itself, is sufficient to alleviate the "ping-pong" effect. In contrast to the previous cost terms, exemplified by the parameters ν and μ, however, this dummy variable does not enforce low firing rates and thus, does not lead to a systematic underestimation of $\mathbf{x}$.

We ran simulations for the homogeneous integrator as in the main paper (i.e. $N = 400$ neurons, 200 with kernel $\mathbf{\Gamma}_i = [0.1, \sqrt{\rho}]$, and 200 with kernel $\mathbf{\Gamma}_i = [-0.1, \sqrt{\rho}]$), with $\rho = 0.0016$, $r_o = 2400$Hz. We used additional linear and quadratic cost terms with $\nu = 0.005$ and $\mu = 0.005$. Note that r_o is a population firing rate corresponding to a mean firing rate of 6 Hz per unit. All other parameters are the same as in the method section. To implement synaptic failure, we randomly multiplied each postsynaptic weight Ω^s_{ij} and Ω^f_{ij} by independent binomial random variables with probability $p = 1 - P/100$ (where P is the percent of synaptic failures) each time a new spike is fired in the network. To keep the average connection strength between two neurons the same, we multiplied the fast and slow connections by $1/p$.

An example simulation of the homogeneous integrator network with 20% synaptic failure is shown in Supplementary Fig. 4E–F. We found that as the percentage of synaptic failures increases, the network fires more spikes, and the prediction error (defined as the time average $\langle (x - \hat{x})^2 \rangle^{0.5}$) increases, see Supplementary Fig. 4F. However, even for high levels of failures (up to 50% in this example), the performance of the network largely exceeds a rate model with the same instantaneous firing rates. The data in Supplementary Fig. 4F were obtained by measuring the mean prediction error and spike counts on repeated presentation of the trajectory in Supplementary Fig. 4D,E. We also tested the network on different trajectories (e.g. c drawn from gaussian white noise) and found similar results.

How does the network correct the fluctuations introduced by synaptic failures? This is illustrated on Supplementary Fig. 5A,B. Here we show the membrane potential and spike trains of a toy example composed of only 4 neurons (same kernels as in Supplementary Fig. 1C). The network in Supplementary Fig. 5A does not have any synaptic failures. The network in Supplementary Fig. 5B suffers from 20% synaptic failures, and enforces a constant baseline rate $r_o = 200Hz$ with $\rho = 0.00025$. In both cases the cost terms are $\mu = 0.0005$, $\nu = 0.0005$. Both networks represent a constant variable $x = 3$.

The network with failures fires more spikes, but tracks the variable reliably. In particular the fast inhibitory connection between neurons 1 and 2 (green and blue) fails periodically and causes both neurons to fire synchronously (grey square on Supplementary Fig. 1B). This in turns recruits neuron 3 or 4 (with negative kernels, magenta or black), which correct these errors. However, in contrast to Supplementary Fig. 1C, this does not lead to a "ping pong" effect.

From the example above we conclude that this framework is successful even with high levels of synaptic failure, provided that the "ping pong effect" can be avoided. This can be achieved by setting the kernels (effectively adding dummy variables) such that they do not point in opposite directions. Note that the reliability of synaptic transmission is detrimental mainly for large inhibitory "fast" connections. Failure in these connections causes synchronous firing between neurons of similar selectivity. This problem can be largely resolved if strong synapses (such as reciprocal synapses with local inhibitory interneurons) were also more reliable. For example, two nearby pyramidal cells in the somatosensory cortex have a very high chance of di-synaptically inhibiting each other. This inhibition recruits a population of interneurons and is very reliable [3].

The network is also robust to strong levels of noise added to the lateral connections. This is illustrated in Supplementary Fig. 5C. In this example we used the homogeneous network with $N = 400$, $\mu = 0.02$, $\nu = 0.02$, $\rho = 0.05$ and $r_o = 2400Hz$ and corrupted the gain of lateral connections by 20%, i.e.we multiplied all lateral connections by a rectified gaussian variable of mean 1 and STD 0.2. Since the connection noise is independent on each connection, these fluctuations are largely averaged out on each neuron, and larger networks become less and less sensitive to such mistuning of the weight. In comparison, global mistuning of the ratio between excitation and inhibition can have a very destructive effect on the stability of the perfect integrator (see Section 4). We found that in this parameter range, the network could tolerate a connection noise of up to 30%.

4 The homogeneous, leaky integrator network: Analytical results

In this section, we study the homogeneous, leaky integrator network for the case without costs. In the article (Figure 1 and Figure 2), we studied the leaky integrator using two populations of neurons, one representing the positive values of x, another representing negative values. For simplicity, we will focus here on a single population of neurons with identical tuning. First, we show that the network implements the required linear dynamical system in the limit of high firing rates.

4.1 Reduction to a single neuron

In the leaky integrator network with identical neurons, all the output kernels are the same so that $\Gamma_i = \Gamma$. In turn, the voltage equations of all neurons are also identical except for the added noise term. The subthreshold voltages are completely synchronized and, for a constant external input, will all rise in unison to threshold. Consequently, random noise is responsible for determining which neuron hits threshold first and fires. From a mathematical point of view, the network is equivalent to a single neuron that is firing in every integration cycle. We will therefore restrict ourselves to the analysis of this single neuron which provides all the information necessary to understand the network dynamics.

This single neuron is supposed to implement the linear dynamical system

$$\dot{x} = -\lambda_s x + c(t) \tag{S23}$$

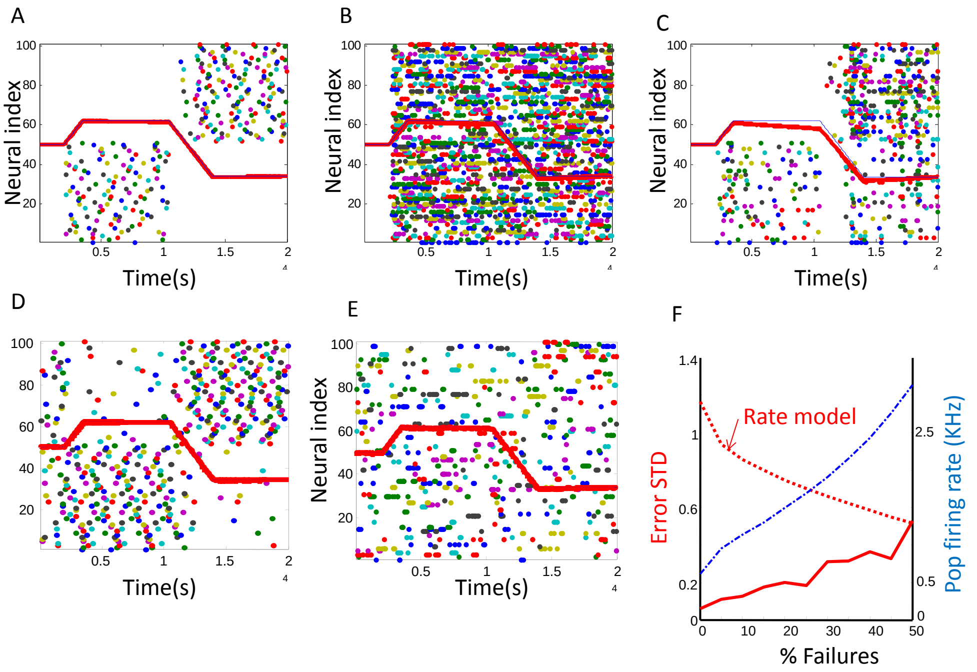

Supplementary Figure 4: Consequences of synaptic failure. **A.** Homogeneous integrator network without synaptic failures (same parameter as in the main text). **B.** Homogeneous integrator with 10% synaptic failure, and costs $\mu = 0.01$ and $\nu = 0.01$. **C.** Homogeneous network with 10% synaptic failure and costs $\mu = 0.03$, $\nu = 0.03$. **D.** Homogeneous network with added constraint on baseline firing rate $\rho = 0.0016$ and $r_0 = 600Hz$ (see text). No synaptic failures. **E.** Same as in (D) but with 20% synaptic failures. **F.** Homogeneous network with added constraint on baseline firing rate ($\rho = 0.0016$ and $r_0 = 600Hz$): Population firing rate (blue dashed line), and mean prediction error (red plain line) as a function of synaptic failure. Dashed red line represents the estimation error of a population of neurons with independent Poisson noise and the same instantaneous firing rate.

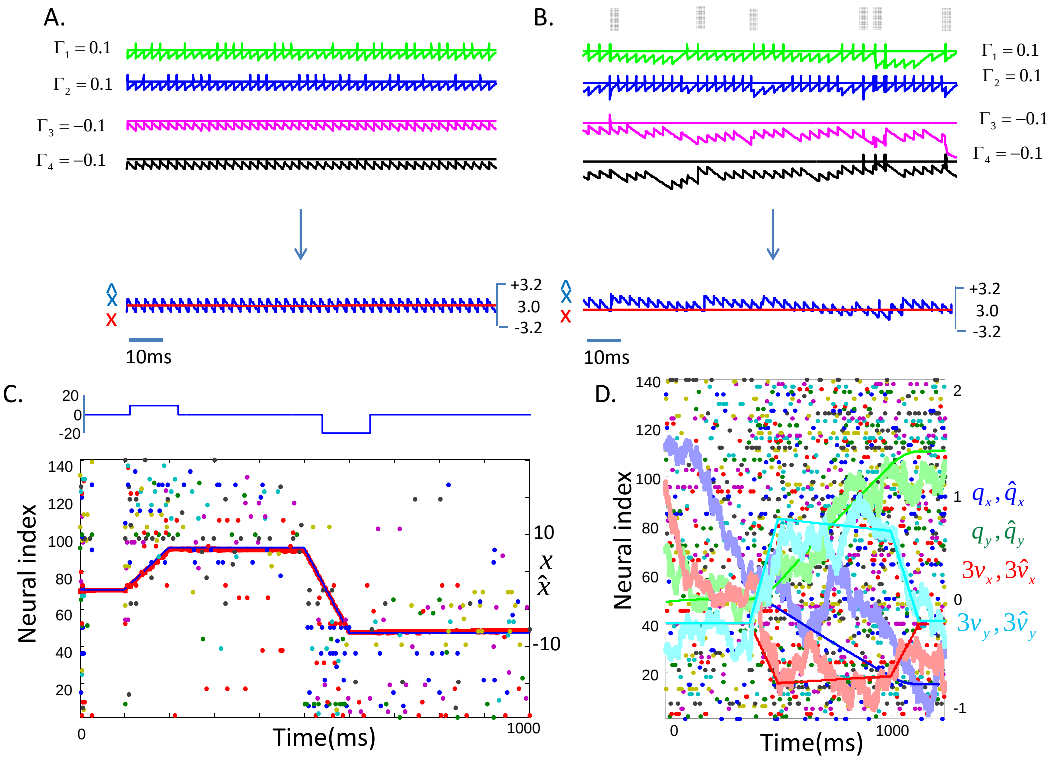

Supplementary Figure 5: Robustness and precision of the network. **A.** Toy example: Homogeneous network with only 4 neurons. In the time slice showed, $x = 3$ and $c = 0$. **B.** Same as in (A) but with 20% synaptic failures in all the connections. **C.** Homogeneous network with $N = 400$ neurons, and added constraint on baseline firing rate ($\rho = 0.0016$, $r_o = 600Hz$) with 20% random noise added to all the connection weights (see text). **D.** Spike trains (dots), arm state (thin lines) and estimates from the linear decoder (thick lines) for the arm controller shown in Fig. 5A in the main text, but with spike trains recorded in different trials.

using its spike train

$$s(t) = \sum_i \delta(t - t_i) \tag{S24}$$

such that the dynamical variable x can be read out according to

$$\dot{\hat{x}} = -\lambda_d \hat{x} + \Gamma s(t) \tag{S25}$$

We note that the latter equation has an analytical solution, given by

$$\hat{x}(t) = \Gamma \int_0^\infty dt' h_d(t') s(t - t') \tag{S26}$$

$$= \Gamma \sum_i h_d(t - t_i) \tag{S27}$$

where

$$h_d(t') = \Theta(t') e^{-\lambda_d t'} \tag{S28}$$

is the integration kernel.[5] Next, we introduce the "normalized" voltage, given by

$$V = \frac{1}{\Gamma}(x - \hat{x}). \tag{S29}$$

This definition differs from the one in the main article by a factor Γ^2, a change we introduce here for notational convenience. With this new definition, the spiking threshold becomes (in the limit $\nu \to 0$)

$$T = \frac{1}{2}. \tag{S30}$$

The neuron's dynamical equation is given by simple derivation of the voltage equation, and self-consistent substitution of x by $\hat{x}$. We obtain

$$\dot{V} = \frac{1}{\Gamma} c(t) + \frac{\mu}{\Gamma} \hat{x}(t) - s(t) \tag{S31}$$

where we have defined

$$\mu = \lambda_d - \lambda_s\,. \tag{S32}$$

Apart from the missing leak term, the voltage equation is the single-neuron variant of Eq. (5) in the main article. The slow recurrent inputs are mediated by $\hat{x}(t)$, as given in Eq. (S27), and the fast recurrent inputs are mediated by the spike train $s(t)$.

4.2 The integrate-and-fire neuron

If a spike occurs, a delta-function is added to the spike train $s(t)$, and, once we integrate Eq. (S31) over the respective spike time, this delta-function causes a decrease of the voltage by 1, thereby effectively resetting the membrane to $V = -1/2$. Consequently, we can simply replace the fast recurrent inputs $s(t)$ in the voltage equation by making this reset explicit. Furthermore, we will also introduce a leak term, thus making the voltage equation similar to an integrate-and-fire neuron,

$$\dot{V} = -\lambda_V V + \frac{1}{\Gamma} c(t) + \frac{\mu}{\Gamma} \hat{x}(t) \tag{S33}$$

[5] Since the spike trains incorporate the complete and infinite past, we do not specify any initial conditions in the solution of the differential equations.

with reset,

$$V \to V_{\text{rest}} = -\frac{1}{2} \tag{S34}$$

after each spike. Note that the leak pulls the neuron towards $V = 0$, and not towards the reset potential. This definition is arbitrary and any other choice of resting potential can be made without affecting the performance of the network.

4.3 Solution of the integrate-and-fire neuron

We can compute the interspike interval of the integrate-and-fire neuron for a given input if we assume that the external inputs $c(t)$ are changing slowly. Directly after a spike at time $t = 0$, the voltage has been reset to $V(0) = -1/2$. We can then integrate the voltage equation, using this initial condition, $V(0) = -1/2$, to obtain

$$V(t) = -\frac{1}{2}e^{-\lambda_V t} + \frac{1}{\Gamma}\int_0^t dt' g(t')c(t-t') + \frac{\mu}{\Gamma}\int_0^t dt' g(t')\hat{x}(t-t') \tag{S35}$$

where $g(t')$ is the integration kernel, given by

$$g(t') = \Theta(t')e^{-\lambda_V t'}. \tag{S36}$$

The first term of the voltage equation is a transient term caused by the initial condition. It appears because the resting potential ($V = 0$) and the reset potential ($V = -1/2$) are not identical. This, however, is simply a matter of definition and non-essential for the proper functioning of the network.

The second term of the voltage equation is due to the filtering of the external inputs $c(t)$ by the membrane. If $c(t)$ varies sufficiently slowly (compared to the average interspike interval), we can safely assume that $c(t)$ will remain approximately constant within each interspike interval. Defining $c(t) \approx c(0) = c_0$, we can rewrite the second term as

$$\int_0^t dt' g(t')c(t-t') = \frac{c_0}{\lambda_V}\left(1 - e^{-\lambda_V t}\right) \tag{S37}$$

The third term is due to the slow synaptic input from the neuron's own (past) spikes. These spikes occurred for times $t_i \leq 0$. Keeping that in mind and plugging $\hat{x}$ from Eq. (S27) into the right-hand integral, we obtain

$$\int_0^t dt' g(t')\hat{x}(t-t') = \Gamma\sum_i \int_0^t dt' \Theta(t')e^{-\lambda_V t'}\Theta(t-t'-t_i)e^{-\lambda_d(t-t'-t_i)} \tag{S38}$$

$$= \Gamma\sum_i e^{-\lambda_d(t-t_i)}\int_0^t dt' e^{-(\lambda_V-\lambda_d)t'} \tag{S39}$$

$$= \Gamma\sum_i e^{-\lambda_d(t-t_i)}\left[\frac{1-e^{-(\lambda_V-\lambda_d)t}}{\lambda_V-\lambda_d}\right] \tag{S40}$$

$$= \Gamma\sum_i e^{\lambda_d t_i}\left[\frac{e^{-\lambda_d t}-e^{-\lambda_V t}}{\lambda_V-\lambda_d}\right] \tag{S41}$$

$$= \hat{x}_0\left[\frac{e^{-\lambda_d t}-e^{-\lambda_V t}}{\lambda_V-\lambda_d}\right] \tag{S42}$$

where in the last step we defined

$$\hat{x}_0 := \hat{x}(0) = \Gamma \sum_{t_i<0} e^{\lambda_d t_i} \tag{S43}$$

If we replace these terms in Eq. (S35), we obtain

$$V(t) = -\frac{1}{2}e^{-\lambda_V t} + \frac{c_0}{\Gamma\lambda_V}\big(1 - e^{-\lambda_V t}\big) + \frac{\mu\hat{x}_0}{\Gamma}\Big[\frac{e^{-\lambda_d t} - e^{-\lambda_V t}}{\lambda_V - \lambda_d}\Big] \tag{S44}$$

which is now simply a function of time t, and of all the parameters entering the model. We note that both the external inputs $c(t)$ and the recurrent inputs $\hat{x}(t)$ are represented through their respective values at time $t = 0$. To obtain the interspike interval, i.e., the timing of the next spike, we need to figure out when the voltage $V(t)$ will hit the threshold, $T = 1/2$. If we denote the time of the next spike as Δt, then

$$\frac{1}{2} = -\frac{1}{2}e^{-\lambda_V \Delta t} + \frac{c_0}{\Gamma\lambda_V}\big(1 - e^{-\lambda_V \Delta t}\big) + \frac{\mu\hat{x}_0}{\Gamma}\left(\frac{e^{-\lambda_d \Delta t} - e^{-\lambda_V \Delta t}}{\lambda_V - \lambda_d}\right). \tag{S45}$$

This equation cannot be solved in closed form for Δt since it includes exponentials with two different time constants. Approximate solutions (e.g. using Taylor expansions of implicit functions) are possible. Here, we will first consider the case of high firing rates (and thereby small interspike intervals) and show that the neuron's spikes reproduce the required dynamics.

4.4 The limit of high firing rates

The firing rate of the neuron we are considering essentially corresponds to the number of integration cycles per time unit that the network is undergoing. As the number of neurons in the network increase, while the firing rates of the neurons remain constant, the number of integration cycles increases. Hence, the large network limit is equivalent to the high firing rate limit in the single neuron case.

If the firing rate of the single neuron is sufficiently high, then the interspike intervals, defined as the reciprocal of the rate, will be quite small. In turn, we can expand the exponentials in the voltage equation, Eq. (S45), to first order,

$$\frac{1}{2} = -\frac{1}{2}(1 - \lambda_V \Delta t) + \frac{c_0}{\Gamma\lambda_V}\lambda_V \Delta t + \frac{\mu\hat{x}_0}{\Gamma}\Delta t. \tag{S46}$$

This equation is easily solved for Δt. If we define the instantaneous firing rate as $r_0 = 1/\Delta t$, i.e., the reciprocal of the interspike interval, then we obtain

$$r_0 = \frac{1}{\Delta t} = \frac{1}{2}\lambda_V + \frac{c_0}{\Gamma} + \frac{\mu\hat{x}_0}{\Gamma} \tag{S47}$$

which yields an equation for the current firing rate as a function of the external inputs and the past spiking activity. In turn, we can use this firing rate to replace the spike trains in the read-out equation, as long as the interspike intervals are small compared to the read-out time constant $1/\lambda_d$. Taking into account that the firing rate r_0 can change over time (once we move beyond the current

interspike interval), we write $r(t)$, and plug this into the read-out equation Eq. (S48) to obtain

$$\frac{d\hat{x}}{dt} = -\lambda_d \hat{x}(t) + \Gamma r(t) \tag{S48}$$

$$= -\lambda_d \hat{x}(t) + \frac{1}{2}\lambda_V \Gamma + c(t) + \mu \hat{x}(t) \tag{S49}$$

$$= -\lambda_s \hat{x}(t) + c(t) + \frac{1}{2}\lambda_V \Gamma \tag{S50}$$

where in the last step, we replaced $\mu = \lambda_d - \lambda_s$ from Eq. (S32). We have therefore retrieved the required dynamical equation up to the term $\lambda_V \Gamma/2$. This term becomes negligible if Γ is sufficiently small (weak synapses) or if λ_V is sufficiently small (slow membrane time constant or negligible leak term) when compared to the size of $\hat{x}$ or $c(t)$. For the homogeneous network, the output kernel Γ will decrease with the network size, thereby guaranteeing that the working regime of the system will be reached.

4.5 Robustness to perturbations: imbalances in excitation and inhibition

These analytical results allow us to study a particularly interesting case of global mistuning of the network. Imagine that for some reason the balance of excitation and inhibition in the network was disturbed. In the homogeneous network, this would mean that the excitatory inputs, either through the feedforward term $c(t)$ or the recurrent term $\hat{x}(t)$, are no longer in balance with the fast inhibitory inputs to the neurons. Note that in the one-neuron version of the network, these inhibitory inputs are mediated through the spike train $s(t)$ in Eq. (S31), which was subsequently replaced by the voltage reset.

To study this scenario, we therefore keep the voltage reset constant, and focus on changes in the excitatory inputs. Let us assume that the parameter $\mu = \lambda_d - \lambda_s$, which determines the strength of the recurrent excitatory input, is perturbed so that $\tilde{\mu} = \mu(1+\epsilon)$ with $|\epsilon| \ll 1$. In turn, even in the limit of high firing rates, the readout Eq. (S48) becomes

$$\frac{d\hat{x}}{dx} = -\lambda_d \hat{x}(t) + \frac{\lambda_V}{2\Gamma} + c(t) + \tilde{\mu}\hat{x}(t) \tag{S51}$$

$$= -\big(\lambda_s - \epsilon(\lambda_d - \lambda_s)\big)\hat{x}(t) + c(t) + \frac{\lambda_V}{2\Gamma} \tag{S52}$$

so that a disturbance in the balance of excitation and inhibition will change the dynamical equation implemented by the network. For a leaky integrator, this may be less than dramatic. For $\lambda_s = 0$, however, such perturbations can be dramatic, since the mistuning of the excitatory inputs then leads to an imperfect integrator that remains workable only for time scales of the order $1/(\lambda_d \epsilon)$. This is the well-known "fine-tuning" problem of integrator systems (see [2] for an elegant solution of this problem). Hence, although the network dynamics are robust against random perturbations of individual neurons, they are not robust against global perturbations of the excitation versus inhibition balance.

References

[1] Brunel N, Nadal J (1998) Mutual information, Fisher information, and population coding. *Neural Comput* 10(7):1731–57.

[2] Moreau L, Sontag E (2003) Balancing at the border of instability. *Phys Rev E* 68: 020901(R).

[3] Silberberg G, Grillner S, LeBeau FE, Maex R, Markram H. (2005) Synaptic pathways in neural microcircuits. *Trends Neurosci* 28(10):541–51
